# Integrative iTRAQ-based proteomic and transcriptomic analysis reveals the accumulation patterns of key metabolites associated with oil quality during seed ripening of *Camellia oleifera*

**DOI:** 10.1038/s41438-021-00591-2

**Published:** 2021-07-01

**Authors:** Zhouchen Ye, Jing Yu, Wuping Yan, Junfeng Zhang, Dongmei Yang, Guanglong Yao, Zijin Liu, Yougen Wu, Xilin Hou

**Affiliations:** 1 College of Horticulture, Hainan University, Haikou, China; 2 State Key Laboratory of Crop Genetics & Germplasm Enhancement, Key Laboratory of Biology and Genetic Improvement of Horticultural Crops (East China), Ministry of Agriculture and Rural Affairs of the P.R. China, Engineering Research Center of Germplasm Enhancement and Utilization of Horticultural Crops, Ministry of Education of the P.R. China, Institute of Plasma Engineering, Nanjing, China

**Keywords:** Transcriptomics, Plant molecular biology

## Abstract

*Camellia oleifera* (*C. oleifera*) is one of the four major woody oil-bearing crops in the world and has relatively high ecological, economic, and medicinal value. Its seeds undergo a series of complex physiological and biochemical changes during ripening, which is mainly manifested as the accumulation and transformation of certain metabolites closely related to oil quality, especially flavonoids and fatty acids. To obtain new insights into the underlying molecular mechanisms, a parallel analysis of the transcriptome and proteome profiles of *C. oleifera* seeds at different maturity levels was conducted using RNA sequencing (RNA-seq) and isobaric tags for relative and absolute quantification (iTRAQ) complemented with gas chromatography-mass spectrometry (GC-MS) data. A total of 16,530 transcripts and 1228 proteins were recognized with significant differential abundances in pairwise comparisons of samples at various developmental stages. Among these, 317 were coexpressed with a poor correlation, and most were involved in metabolic processes, including fatty acid metabolism, α-linolenic acid metabolism, and glutathione metabolism. In addition, the content of total flavonoids decreased gradually with seed maturity, and the levels of fatty acids generally peaked at the fat accumulation stage; these results basically agreed with the regulation patterns of genes or proteins in the corresponding pathways. The expression levels of proteins annotated as upstream candidates of phenylalanine ammonia-lyase (PAL) and chalcone synthase (CHS) as well as their cognate transcripts were positively correlated with the variation in the flavonoid content, while shikimate O-hydroxycinnamoyltransferase (HCT)-encoding genes had the opposite pattern. The increase in the abundance of proteins and mRNAs corresponding to alcohol dehydrogenase (ADH) was associated with a reduction in linoleic acid synthesis. Using weighted gene coexpression network analysis (WGCNA), we further identified six unique modules related to flavonoid, oil, and fatty acid anabolism that contained hub genes or proteins similar to transcription factors (TFs), such as MADS intervening keratin-like and C-terminal (MIKC_MADS), type-B authentic response regulator (ARR-B), and basic helix-loop-helix (bHLH). Finally, based on the known metabolic pathways and WGCNA combined with the correlation analysis, five coexpressed transcripts and proteins composed of cinnamyl-alcohol dehydrogenases (CADs), caffeic acid 3-O-methyltransferase (COMT), flavonol synthase (FLS), and 4-coumarate: CoA ligase (4CL) were screened out. With this exploratory multiomics dataset, our results presented a dynamic picture regarding the maturation process of *C. oleifera* seeds on Hainan Island, not only revealing the temporal specific expression of key candidate genes and proteins but also providing a scientific basis for the genetic improvement of this tree species.

## Introduction

*Camellia oleifera* (*C. oleifera*), native to East Asia, is a valuable oilseed crop belonging to the genus *Camellia* of the Theaceae family^1^. It has been cultivated for more than 1000 years, with an annual output of seeds exceeding 2.4 million tons distributed over an area of 4.4 million hectares in China^2^. As an economically important tree species, its seeds can be pressed to yield edible oil that is rich in unsaturated fatty acids (UFAs, e.g., oleic acid, linoleic acid, and linolenic acid) as well as natural bioactive ingredients (e.g., squalene, sterols, flavonoids, and tocopherols) and that complies completely with the international nutritional standards of “omega meals”^3^. This oil has been recorded in the Chinese Pharmacopoeia as having health-promoting effects on scavenging free radicals, lowering blood pressure, delaying atherosclerosis, reducing cholesterol, and improving immunity^4^. Thus, *C. oleifera* oil is considered to be an attractive raw material and functional product in the food industry and has been widely used worldwide.

In particular, *C. oleifera* on Hainan Island has experienced long-term geographic isolation from the mainland and is regarded as an independent population and a traditional plant resource. The unique climatic conditions and hereditary characteristics have given birth to an abundant and distinctive *C. oleifera* cultivated species with a large amount of genetic variation^5^. Our previous studies have demonstrated that the content of certain nutrient substances in this cultivar is higher than that in other varieties, especially flavonoids and fatty acids^6^. Their synthesis pathways may be regulated both spatially and temporally during seed ripening, and these pathways are highly coordinated genetic programming processes involving the expression of numerous genes that can be analyzed by Illumina RNA sequencing (RNA-seq)^7^. For example, chalcone isomerase (CHI) has been recognized as a rate-limiting enzyme in the catechin-producing branch^8^; omega-6 fatty acid desaturase-2 has been demonstrated to be able to desaturate oleic acid to generate linoleic acid^9^; and long-chain acyl-CoA synthetase (LACS) has been shown to catalyze the formation of free fatty acids from acyl-CoA^10^. However, the transcriptomic dataset alone is insufficient for fully understanding the biosynthetic network because it only represents the mRNA expression level but does not take into account the presence of posttranslational modifications^11^. Most of the gene functions are ultimately realized in the form of proteins that are thought to have a more direct relationship with metabolites^12^.

Proteomics, which is a large-scale study of protein structure and function, can serve as an effective tool for obtaining information concerning specific biological reactions and as such is a powerful technique for identifying the proteins responsible for regulating metabolic pathways involved in seed growth and development^13,14^. Many comparative proteomics studies have been conducted on higher plants, with methods consisting of two-dimensional gel electrophoresis, difference gel electrophoresis, and label-free shotgun^15^. Surprisingly, the newly developed isobaric tags for relative and absolute quantification (iTRAQ) labeling technology, which can quantify protein levels with a higher accuracy, has been confined to a small number of species, including tomato^16^, peach^17^, blood orange^18^, and oriental melon^19^. Therefore, we believe that iTRAQ-based quantitative proteome analysis of *C. oleifera* would greatly enhance our understanding of its seed maturation process. Moreover, the field of potentially critical genes or proteins could be narrowed by creating modules based on weighted gene coexpression network analysis (WGCNA) with the data generated via RNA-Seq or iTRAQ assays. This promising approach has been shown to be effective in identifying the modules of coexpressed genes or proteins, as well as in correlating these distinct modules with phenotypic traits, to further detect the key genes (proteins) within the networks and understand their regulatory mechanisms in living systems^20,21^.

In light of the above, a complementary analysis was carried out to acquire global proteome and transcriptome datasets of *C. oleifera* seeds at different levels of maturity using iTRAQ and RNA-seq methodologies complemented by metabolic results. Then, a gene coexpression network was constructed based on WGCNA and combined with correlation analysis to further screen out the core genes or proteins. Finally, quantitative real-time PCR (qRT-PCR) was performed for 31 pivotal coexpressive transcripts and proteins to validate their changes in abundance. The current study aimed to (i) gain a broader systematic view of dynamic alterations in central metabolism at various stages of *C. oleifera* seed development and ripening; (ii) provide a detailed framework for the practical association and difference between transcriptomic and proteomic profiles; and (iii) identify a set of key candidate genes and proteins related to flavonoid and fatty acid anabolism pathways and investigate their potential coordinated regulatory mechanisms. In addition, the findings presented herein may lay the foundation for preliminarily characterizing the complex molecular networks controlling metabolite accumulation processes of oil-bearing crops and expanding the exploitation and utilization of interspecific resources within the same genus.

## Materials and methods

### Plant materials

Fresh fruits of *C. oleifera* were harvested in 2018 from Yangjiang town (19° 12′ 10″ N; 110° 24′ 32″ E), Qionghai city, Hainan Province, China. Four developmental periods were sampled from August to November: the nutrition synthesis stage (S1), fat accumulation stage (S2), mature stage (S3), and late mature stage (S4). The growth conditions, selection criteria, and sampling method for the plants followed those previously described^22^. For each phase, uniform fruits were pooled and divided into quarters for transcriptome sequencing, proteome profiling, metabolite detection, and qRT-PCR verification. Therefore, one-half of the samples were flash-frozen under liquid nitrogen after peeling and wrapping in tinfoil and then stored at −80 °C for later analysis, while the other half were air-dried for assays of physicochemical properties.

### Measurement of physiological parameters

Total flavonoids were determined according to a colorimetric method reported in the literature^23^, and the content was recorded in units of micrograms rutin equivalents (mg RE/g) based on a standard calibration curve. The amount of phenylpropanoid was measured at a 740 nm wavelength via the procedure developed by Xin et al.^24^, using α-asarone as a reference. In addition, the fatty acid composition was detected through a protocol that was set in accordance with Chinese Standard GB 5009.168-2016. The standard preparation, sample pretreatment, and gas chromatography-mass spectrometry (GC-MS) determination conditions were described in our previous paper^22^. All samples for metabolite identification were analyzed in triplicate, and the data are presented as the means ± standard deviation (SD). The statistical significance of physiological characteristics was evaluated by one-way analysis of variance (ANOVA) with Duncan’s multiple comparison test (*p* < 0.05) in IBM SPSS_v.19.0.

### Transcriptome profiling

Total RNA was isolated from *C. oleifera* seeds using TRIzol Universal Reagent (Tiangen Biotech, China) in accordance with the manufacturer’s recommendations. Sequencing libraries were constructed using the NEBNext^®^ UltraTM RNA Library Prep Kit for Illumina^®^ (NEB, USA) by Biotree Biomedical Technology Co., Ltd (Shanghai, China). Briefly, mRNA was enriched by oligo (dT) beads and decomposed by fragmentation buffer. These short fragments were reverse-transcribed into cDNA using random hexamer primers, and second-strand complementary DNA (cDNA) was subsequently synthesized using dNTPs, DNA polymerase I, RNase H, and buffer. Finally, the ligated products were selected by agarose gel electrophoresis, PCR amplified, and then sequenced on an Illumina NovaSeq platform. Next, clean reads were obtained by removing unqualified reads with ambiguous nucleotides, and adapter sequences were filtered from raw reads. After de novo assembly via Trinity software, the abundance of unigenes was estimated from the read counts and normalized as FPKM (expected number of fragments per kilobase of transcript sequence per million base pairs sequenced). The relative expression level of each transcript was calculated by the statistical package DEGseq2, and the resulting *p* values were adjusted by controlling for the false discovery rate (FDR). Genes with |log_2_-fold change| > 1 and *p* adj < 0.05 were considered differentially expressed genes (DEGs)^25^. Functional annotation of unigenes was carried out by BLASTx searches of the following public databases: Nr (NCBI nonredundant protein sequences), Nt (NCBI nonredundant nucleotide sequences), Pfam (Protein family), KOG (euKaryotic Orthologous Groups of proteins), Swiss-Prot (Swiss-Prot protein sequence database), KO (Kyoto Encyclopedia of Genes and Genomes Ortholog database), and GO (Gene Ontology), with a threshold *E* value of 10^−5^.

### Proteome profiling

#### Protein extraction and quantitative analysis

Total seed protein was extracted as described elsewhere with slight modifications^26^. In short, samples were finely ground to a powder with liquid nitrogen in the presence of polyvinylpolypyrrolidone (PVPP) and suspended in a two-phase system consisting of fresh extraction buffer and chilled phenol buffered with Tris(hydroxymethyl)aminomethane hydrochloride (Tris-HCl), pH 7.8. After centrifugation at 7100 × *g* for 10 min at 4 °C, the phenol-based upper phase was transferred to a new conical tube. Then, the protein was precipitated by adding five volumes of precooled methanolic 0.1 M ammonium acetate and incubated at −20 °C overnight. The precipitates were collected and washed with ice-cold methanol and acetone to remove interfering compounds. Next, each pellet was solubilized in sodium dodecyl sulfate (SDS) lysis buffer at room temperature for approximately 3 h. The final protein solution was quantified by using a Bovine Serum Albumin Protein Assay Kit (Thermo Fisher, USA) and confirmed with SDS-polyacrylamide gel electrophoresis (SDS-PAGE).

#### In-solution trypsin digestion and iTRAQ labeling

Protein was digested according to the filter-aided sample preparation protocol as described previously^27^. For each sample, 100 μg of protein was placed on an ultrafiltration filter (10 kDa cutoff) containing 120 μL of reducing buffer [100 mM triethylammonium bicarbonate, 8 M urea, 100 mM dithiothreitol, pH 8.0] and incubated at 60 °C for 1 h. Next, iodoacetamide was incorporated to block any reduced cysteine residue. The mixture was then kept at room temperature for 40 min in darkness followed by centrifugation for 20 min at 15,000 × *g* and 20 °C. In-solution digestion with sequence-grade modified trypsin at 37 °C was performed for 12 h. Thereafter, the resulting peptides were collected in the form of filtrates and labeled using 8-plex iTRAQ reagents (ABSCIEX, USA), following the instructions of the manufacturer (113, 114, 115, and 116 for S1, S2, S3, and S4, respectively, and S1 was used as the control). Three independent biological experiments were conducted. Ultimately, all tagged peptides were multiplexed and vacuum-dried for further identification.

#### SCX fractionation and LC–MS/MS analysis

After labeling, the peptide mixture was fractionated on an Agilent 1100 high-performance liquid chromatography (HPLC) series system (Agilent Technologies, USA) equipped with an Agilent Zorbax Extend-C18 column (2.1 mm × 150 mm, 5 μm). Buffer A was 98% HPLC water with 2% acetonitrile, and buffer B contained 90% acetonitrile with 10% HPLC water. The gradient for separation was generated at a flow rate of 300 nL/min as follows: 98% buffer A for 8 min, 98–95% buffer A for 0.01 min, 95–75% buffer A for 39.99 min, 75–60% buffer A for 12 min, 60–10% buffer A for 0.01 min, 10% buffer A for 9.99 min, 10–98% buffer A for 0.01 min, and 98% buffer A for 4.99 min. The column was re-equilibrated to attain its initial highly aqueous solvent composition prior to analysis. The absorbances at 210 and 280 nm were monitored. The eluent was collected every minute, and 15 fractions were finally pooled according to the chromatogram.

Fractions were then analyzed by using a Q Exactive HF Mass Spectrometer coupled with an Easy-nLC 1200 HPLC system (Thermo Fisher Scientific, USA). The labeled peptides were loaded onto an Acclaim PepMap100 column (RP-C18, 100 μm × 20 mm) using an autosampler. Chromatographic separation was performed with an Acclaim PepMap RSLC column (75 µm × 15 cm). The mobile phases consisted of solvent A (0.1% formic acid in HPLC water) and solvent B (0.1% formic acid, 19.9% HPLC water, 80% acetonitrile). Tryptic peptides were eluted by application of a linear gradient comprising 0–1 min from 2% to 9% solvent B, 1–45 min from 9% to 29% solvent B, 45–52 min from 29% to 37% solvent B, 52–56 min from 37% to 100% solvent B, and 100% solvent B for 4 min.

The mass spectrometer was operated in the data-dependent acquisition mode, wherein the resolution of the full MS scan was set to 60,000, the highest ion injection time was 50 ms, and the automatic gain control (AGC) target was 3e6. Precursor ions were acquired across a mass range of 350–1500 *m*/z, and up to 10 of the most abundant precursors per cycle from each MS spectrum were selected with a 30-s dynamic exclusion duration for subsequent higher-energy collisional dissociation fragment analysis at a collision energy of 30%. The MS/MS spectra were recorded in the high-resolution mode of 15,000, a maximum injection time of 40 ms, and an AGC value of 2e5, with the rolling collision energy on and iTRAQ reagent collision energy adjustment on.

#### Database search, protein quantification, and bioinformatics analysis

For protein identification, raw data were analyzed using the MASCOT search engine embedded in Proteome Discoverer 2.3 software with our above transcriptome database on the basis of sequence homology. The parameters were as follows: static modifications of the iTRAQ 8plex at lysine (Lys), tyrosine (Tyr), the N-terminal amino group of peptides and the carbamidomethyl at cysteine (Cys); dynamic modifications of oxidation at methionine (Met) and the acetyl at the N-terminal amino group of peptides; enzyme specificity was set to trypsin with two missed cleavages; and the mass tolerance was 10 ppm for precursor ions and 0.02 Da for fragmented ions. Proteins that contained at least two unique peptide matches with confidence intervals higher than 95% and FDR values less than 1% were qualified for subsequent quantification analysis^28^. Furthermore, protein species with fold change > 1.2 and *p* value < 0.05 present in not less than two replicates were considered differentially abundant proteins (DAPs). Sequences of the positively identified proteins were employed for BLAST searching against the UniProt database (*E* value = 10^−5^). GO and KEGG enrichment analyses were conducted to determine the functional subcategories and metabolic pathways in which the proteins were significantly enriched. The probable interacting partners between proteins were then further predicted according to the STRING database.

### WGCNA for identifying correlated gene and protein networks

WGCNA was performed using a freely accessible R package with default parameters according to the protocol to recognize coexpressed genes and proteins^29^. WGCNA network construction and module detection were conducted by using an unsigned type of topological overlap matrix, soft-thresholding powers of 30 (genes) and 14 (proteins), a minimum module size of 20, and a branch merge cut height of 0.25. Next, the transcripts or proteins with identical patterns of expression were grouped into one module, and their eigengenes were also calculated. Finally, the phenotype data were imported into the WGCNA software package to obtain correlation-based associations between phenotypes and gene (protein) modules.

### Integrated transcriptome and proteome analysis

The proteins and corresponding transcripts were considered to be correlated if they were both expressed at the same stage. Based on the log_2_-fold change of DEGs and fold change of DAPs, the Spearman correlation coefficients and associated *p* values were calculated, the correlation plots of three comparative analyses (S2 vs. S1, S3 vs. S1, and S4 vs. S1) were also drawn. GO term annotation and KEGG pathway enrichment analysis were then visualized. Moreover, to better understand the regulatory status of the genes and proteins involved in flavonoid and fatty acid anabolism processes, the cognate DEGs and DAPs were mapped to the reference pathways in the KEGG database^30^. DNAMAN software was used to perform amino acid multiple sequence alignment of the key candidate proteins. A phylogenetic tree was rooted via MEGA 6.0 software based on the neighbor-joining method. Protein subcellular localization was conducted by using WoLF PSORT online with 500 bootstrap replications. The motifs of the protein sequences were predicted by Multiple EM for Motif Elicitation online.

### Validation by qRT-PCR analysis

A total of 31 candidate genes related to flavonoid and fatty acid anabolism were screened for qRT-PCR assay. In brief, new RNA was extracted as described above, and then cDNA was synthesized using a RevertAid First Strand cDNA Synthesis Kit (Thermo Fisher Scientific, USA). The gene-specific primer pairs were designed by Primer 5.0 (Premier Biosoft, USA). qRT-PCR was carried out on a LightCycler 96 (F. Hoffmann-La Roche Ltd, Switzerland). The thermal profile consisted of 95 °C for 30 s followed by 40 cycles of 95 °C for 10 s, 55 °C for 30 s, and 72 °C for 30 s, with a final extension step of 72 °C for 30 s. The 2^−ΔΔCt^ method was used to calculate relative changes in gene expression levels, and glyceraldehyde-3-phosphate dehydrogenase (GAPDH) served as an endogenous control for normalization of cycle threshold values. Data are presented as the mean ± SD of three independent biological replicates^31^.

## Results

### Changes in the physiological characteristics of developing *C. oleifera*

**Fig. 1:**
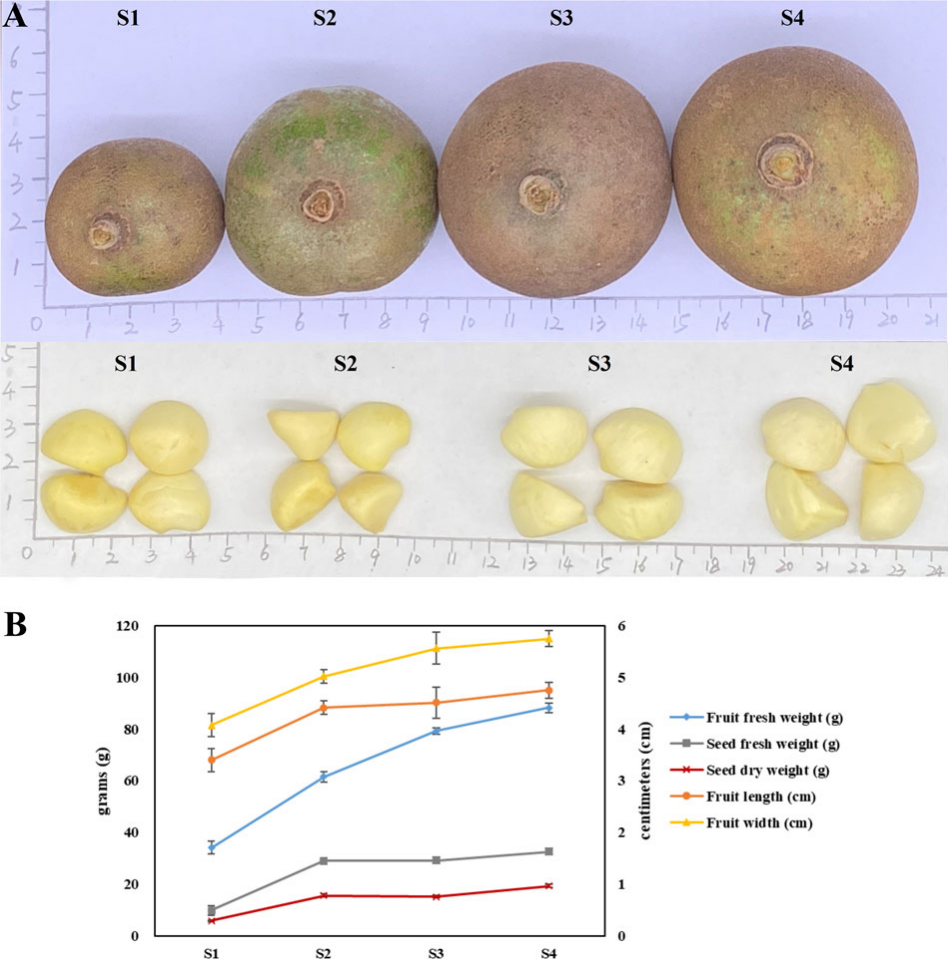
Development of *C. oleifera* seeds. **A** Phenotypic characterization of *C. oleifera* seeds in four growth periods. S1, nutrition synthesis stage; S2, fat accumulation stage; S3, mature stage; and S4, late mature stage. **B** Changes in morphological indexes of developing fruits and seeds. Data represent the mean values from three biological replicates, and error bars indicate standard deviations

The phenotypic characteristics and dynamic changes in reserve accumulation in fruits and seeds during four developmental periods were measured (Fig. 1). The fresh fruits exhibited a gradual increase in size and weight with maturity, and a slight decrease in the dry weight of the seeds was observed at the S3 stage. The oil content (ratio in dry seeds) changed insignificantly from the S1 to S2 stages and then rose rapidly up to the S4 stage. Table 1 shows the composition and amounts of major components in *C. oleifera* samples. With the increase in seed maturity, the concentration of the total flavonoids declined significantly. The S2 stage possessed the highest content of phenylpropanoid, followed by the S1 stage. Moreover, nine common fatty acid compounds were shared among all oil samples; their predominant constituents were similar, being composed of palmitic acid, oleic acid, and linoleic acid together accounting for 96.0% of the total fatty acid profile. The contents of palmitic acid, oleic acid, linoleic acid, and linolenic acid in the S1 and S2 stages were higher than those in other stages. Notably, the highest levels of monounsaturated fatty acids (MUFAs) and polyunsaturated fatty acids (PUFAs) belonged to the S2 and S1 stages, respectively. Nevertheless, the highest ratio of oleic acid to linoleic acid was found in the S4 stage. The total ionization chromatogram of fatty acid methyl ester standards is presented in Fig. S1, and their regression equations are listed in Table S1.

**Table 1:** The content of the main functional components of *C. oleifera* samples at different maturities

Physiological characteristics	S1	S2	S3	S4
Phenylpropanoid content (mg/g)	4.021 ± 0.041^a^	4.028 ± 0.049^a^	3.895 ± 0.014^b^	3.886 ± 0.081^b^
Total flavonoid content (mg/g)	6.821 ± 0.060^a^	5.349 ± 0.010^b^	4.631 ± 0.000^c^	3.401 ± 0.026^d^
Oil content (%)	25.523 ± 0.837^c^	28.971 ± 0.474^b^	41.244 ± 0.223^a^	42.012 ± 0.937^a^
Fatty acid content (g/100 g)				
Palmitic acid (C16:0)	10.799 ± 0.094^a^	9.607 ± 0.063^b^	9.187 ± 0.059^c^	7.905 ± 0.022^d^
Palmitoleic acid (C16:1)	0.083 ± 0.002^a^	0.051 ± 0.002^b^	0.036 ± 0.001^c^	0.026 ± 0.001^d^
Margaric acid (C17:0)	0.035 ± 0.001^b^	0.041 ± 0.000^a^	0.015 ± 0.000^d^	0.025 ± 0.001^c^
Stearic acid (C18:0)	1.815 ± 0.017^d^	2.232 ± 0.022^a^	2.057 ± 0.010^b^	1.931 ± 0.015^c^
Oleic acid (C18:1)	62.921 ± 0.393^b^	68.514 ± 0.328^a^	60.888 ± 0.116^c^	54.689 ± 0.242^d^
Linoleic acid (C18:2)	11.041 ± 0.061^a^	9.600 ± 0.049^b^	8.884 ± 0.063^c^	6.662 ± 0.016^d^
Linolenic acid (C18:3)	0.830 ± 0.005^a^	0.565 ± 0.003^b^	0.416 ± 0.006^c^	0.266 ± 0.003^d^
Arachidic acid (C20:0)	nd	0.025 ± 0.000	nd	nd
Eicosenic acid (C20:1)	0.466 ± 0.006^a^	0.469 ± 0.004^a^	0.426 ± 0.011^b^	0.360 ± 0.002^c^
Tetracosanoic acid (C24:0)	0.079 ± 0.001	0.062 ± 0.001	nd	nd
Tetracosenic acid (C24:1)	0.081 ± 0.001^a^	0.047 ± 0.001^b^	0.035 ± 0.000^c^	0.017 ± 0.000^d^
SFAs	12.650 ± 0.083^a^	11.880 ± 0.084^b^	11.258 ± 0.069^c^	9.862 ± 0.024^d^
MUFAs	63.551 ± 0.398^b^	69.082 ± 0.331^a^	61.385 ± 0.127^c^	55.091 ± 0.239^d^
PUFAs	11.870 ± 0.063^a^	10.164 ± 0.046^b^	9.301 ± 0.057^c^	6.928 ± 0.016^d^
MUFAs/PUFAs	5.354 ± 0.006^d^	6.796 ± 0.011^b^	6.600 ± 0.028^c^	7.952 ± 0.048^a^
Oleic acid/linoleic acid	5.699 ± 0.004^d^	7.137 ± 0.013^b^	6.853 ± 0.036^c^	8.209 ± 0.048^a^

S1, nutrition synthesis stage; S2, fat accumulation stage; S3, mature stage; and S4, late mature stage. Each value is expressed as the mean ± standard deviation; different small letters within a row indicate significant differences (*p* < 0.05)

*nd* not detected

### Transcriptomics analysis

#### Illumina paired-end sequencing and functional annotation of unigenes

After a stringent quality evaluation and data filtering, a total of 709.23 million clean reads (106.38 Gb high-quality sequences) were retained, ranging from 8.02 to 11.49 Gb per sample (Table S2). Using Trinity de novo assembly, all high-quality reads were mutually aligned and assembled into 502,269 transcripts, with lengths between 301 and 80,014 bp (N50 value of 1187 bp). These transcripts were further clustered based on nucleotide sequence identity, resulting in 170,891 unigenes (N50 value of 1106 bp) that included 12,319 unigenes (7.21%) with lengths greater than 2 kb. High correlations were observed among biological replicates (Fig. S2), suggesting that the experiment had good reproducibility and reliability. In total, 137,753 coding sequences were extracted from BLASTx and ESTScan results, which were then searched against the Nr, Nt, Swiss-Prot, Pfam, KO, KOG, and GO databases (Fig. S3 and Table S3). Among them, 28,328 (41.7%) unigenes showed high similarity with sequences of *Actinidia chinensis* var. *chinensis*, and 4623 unigenes had good matches with genes from *Vitis vinifera*, followed by *Quercus suber*. The top-hit species distribution is depicted in Fig. S3 and Table S4. In addition, only a small proportion of unigenes (10,466, 6.12%) carrying protein domains with KOG annotations were subdivided into 25 clusters based on their main biological activities (Fig. S3 and Table S4). The largest categories included posttranslational modification, protein turnover, chaperones (O, 13.70%), and general function prediction only (R, 11.10%).

GO classification was used to describe the properties of gene products in terms of their associated biological processes, cellular components, and molecular functions, among which 46,315 unigenes were categorized into 56 functional subclasses (Fig. S4 and Table S4). The largest number of annotations was in biological processes, where the major GO term was cellular process. At the cellular component level, the predominant group was cell. Binding and catalytic activity were the most representative molecular function categories. The results indicated that these unigenes were responsible for fundamental biological regulation and metabolism common to plants. Pathway-based analysis can assist in understanding the functions and interactions of genes. In the current work, 20,546 unigenes were assigned to biological pathways in the KEGG database, and the most highly represented pathways were carbohydrate metabolism, translation, and folding, sorting, and degradation (Fig. S5 and Table S4).

#### Differences in gene expression patterns during seed ripening and enrichment analysis

**Fig. 2:**
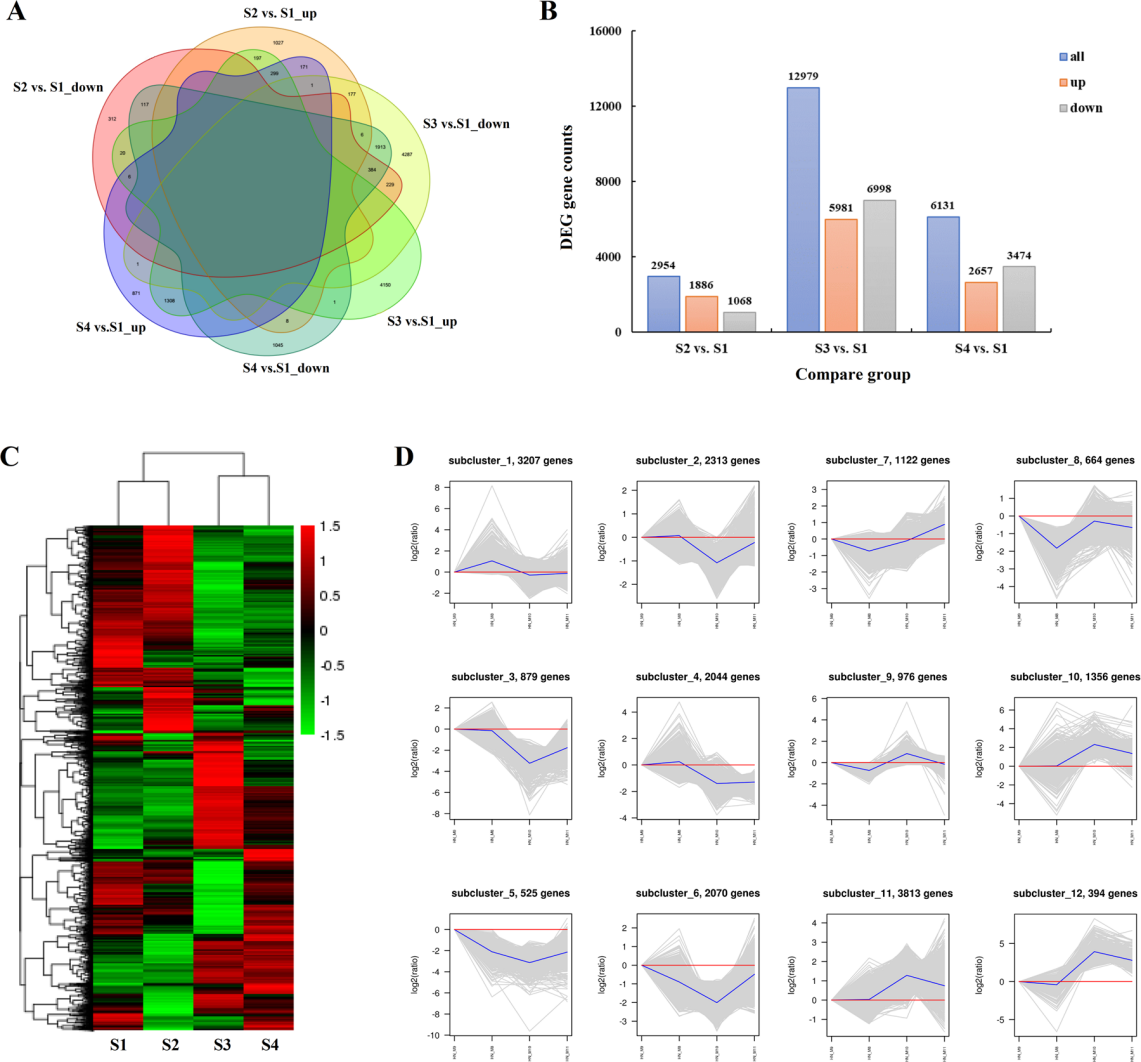
Expression analysis and quantitative comparison of the identified DEGs in developing *C. oleifera* seeds. **A** Venn diagram of the shared and unique DEGs among three compared pairs (S2 vs. S1, S3 vs. S1, S4 vs. S1, S1 as the control). **B** Numbers of up- and downregulated unigenes in different comparisons. **C** Hierarchical clustering analysis of the identified DEGs across four growth periods of seeds. The horizontal axis represents the sample clusters, and colors from green to red indicate gene expression from low to high. **D** The expression trends of the identified DEGs. Gene abundance is expressed as log_2_-fold change (*y*-axis), and developmental stages are outlined on the *x*-axis, with the S1 stage as the zero point

**Fig. 3:**
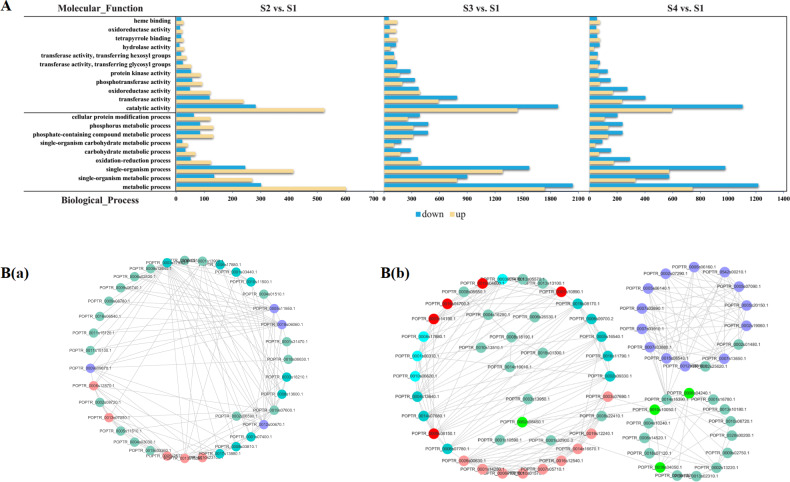
GO-based functional classification and protein–protein interactions of the identified DEGs in developing *C. oleifera* seeds. **A** Top 20 GO categories for the identified DEGs in the transcriptome. Bar diagrams indicate the number of DEGs that were up- and downregulated (*x*-axis), annotated with functions (*y*-axis) for different compared groups. **B** Interaction networks among the predicted unique proteins involved in flavonoid biosynthesis (a) and fatty acid metabolism (b) pathways. The network nodes represent proteins, and the edges represent predicted functional associations between two proteins. Detailed information on protein names and abbreviations is found in Table S7

In total, 16,530 DEGs were identified by pairwise comparison of samples at the four growth periods. Compared with stage S1, 1886 (1068), 5981 (6998), and 2657 (3474) DEGs were significantly upregulated (downregulated) in the S2, S3, and S4 stages, respectively. In addition, only 696 DEGs were detected in all three compared pairs (Figs. 2A, 3B and Table S5). In the results of DEG grouping and sorting by hierarchical clustering analysis, S1 and S2 were classified into one cluster, while S3 and S4 were clustered together according to the relatively high similarity in color (Fig. 2C). Broken line graphs (Fig. 2D) were drawn to classify the expression patterns of DEGs, and the number ascribed to each cluster was also recorded. Cluster I contained 3882 unigenes with the highest initial expression levels and then gradually decreased to the S3 stage. The members of Cluster III showed marked changes in expression, increasing sharply to a maximum at the S2 stage and then declining dramatically at the S3 stage before being slightly upregulated at the S4 stage. For Clusters V and VIII, the expression abundances of unigenes first increased and subsequently declined before reaching their peak values at the S3 stage. The log_2_-fold change of Cluster VIII (approximately −4) was greater than that of Cluster V.

DEGs were further classified into 113 subsets using GO enrichment-based cluster analysis to provide potential clues concerning the molecular events related to their functional roles during seed ripening (Figs. 3A and S6, Table S6). For three pairs of developmental stages, DEGs associated with metabolic process and oxidation–reduction process were overrepresented in the biological process category; catalytic activity was the predominant classification in the molecular function category; and the most assigned classification was cell wall in the cellular component category. The KEGG enrichment analysis of these DEGs could potentially yield information for understanding the molecular mechanisms underlying major metabolic processes in *C. oleifera* seeds (Fig. S7 and Table S6). The pathways with the majority of entries mapped in all comparative analyses included glutathione metabolism, phenylpropanoid biosynthesis, and flavonoid biosynthesis. Other important pathways involving fatty acid degradation, fatty acid biosynthesis, α-linolenic acid metabolism, and linoleic acid metabolism were only found in S3 vs. S1 and S4 vs. S1 pairwise comparisons.

#### Identification and interaction network analysis of flavonoid and fatty acid anabolism-related DEGs, including TFs

We obtained 115 DEGs involved in the phenylpropanoid biosynthesis pathway. Moreover, 37, 9, and 4 DEGs were detected in the flavonoid biosynthesis, flavone and flavonol biosynthesis, and isoflavonoid biosynthesis pathways, respectively. Of these, ten unigenes including peroxidases, ferulate-5-hydroxylase (F5H), and shikimate O-hydroxycinnamoyltransferase (HCT) were markedly upregulated and six unigenes composed of HCTs, flavanone 7-O-glucoside 2″-O-beta-L-rhamnosyltransferase (C12RT1), leucoanthocyanidin reductase (LAR), and beta-glucosidases (bglXs) were downregulated in all comparison analyses, revealing that the HCTs displayed a mixed expression pattern. Specifically, 169 DEGs similar to TFs belonging to the WRKY, basic helix-loop-helix (bHLH), basic region-leucine zipper (bZIP), v-myb avian myeloblastosis viral oncogene homolog (MYB-like), and MADS-box families may be related to the synthetic regulation of flavonoids. Six WRKY-related genes and one unigene encoding MYB were significantly upregulated in three pairwise comparisons, while nine unigenes were downregulated. Concurrently, we found 39, 14, and 53 candidate DEGs associated with the pathways of fatty acid biosynthesis, fatty acid elongation and fatty acid degradation, respectively. In addition, 26 and 58 DEGs were identified in the linoleic acid metabolism and α-linolenic acid metabolism pathways, respectively. Only one key unigene (3-ketoacyl-CoA synthase, KCS) therein was continuously upregulated in the pairwise comparisons. Remarkably, 129 DEGs annotated as members of the DNA binding with one finger (DOF), homeodomain leucine zipper (HD-ZIP), APETALA2 (AP2), and B3 TF families were also involved in fatty acid metabolism processes, among which nine AP2 genes were upregulated in all three groups (Table S6).

Subsequently, a protein–protein interaction (PPI) network was constructed to predict the putative functions and relationships of the identified DEGs. The comprehensive analysis showed that among the 137 DEGs related to flavonoid biosynthesis, 56 DEGs (19 unique proteins) appeared to be closely linked and were classified into three subclusters, with the seed proteins being dihydroflavonol 4-reductase (DFR), 4-coumarate: CoA ligase (4CL), and cinnamyl-alcohol dehydrogenase (CAD). Similarly, among the 137 DEGs involved in fatty acid metabolism, 109 DEGs (30 unique proteins) exhibited strong interactions and were present in six subclusters whose seed proteins were alcohol dehydrogenase (ADH), acetyl-CoA carboxylase/biotin carboxylase 1 (ACACA), acetyl-CoA C-acetyltransferase (ACAT), long-chain acyl-CoA synthetase (ACSL), [acyl-carrier-protein] S-malonyltransferase (fabD), and 3-hydroxyacyl-[acyl-carrier-protein] dehydratase (fabZ) (Fig. 3B).

#### Analysis of the gene coexpression network

**Fig. 4:**
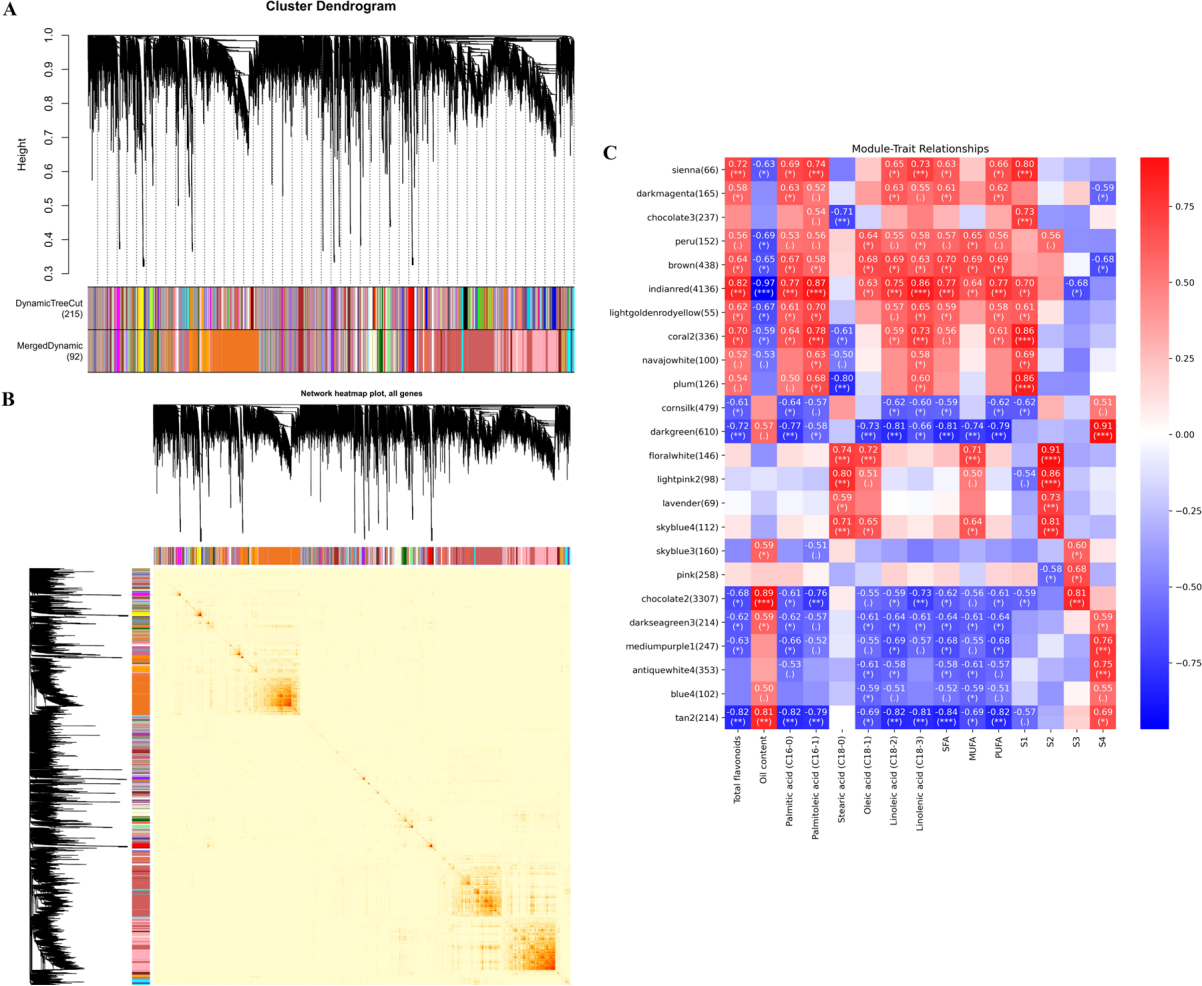
Weighted gene coexpression network analysis (WGCNA) of the identified genes in developing *C. oleifera* seeds. **A** Gene dendrogram obtained by clustering the dissimilarity based on consensus topological overlap, with each tree branch constituting a module and each leaf representing one gene. Each colored row indicates a color-coded module that contains a group of highly interconnected genes. **B** Heatmap plot of topological overlap in the gene network. Darker squares along the diagonal correspond to modules. **C** Module eigengene physiological indexes and sample correlations. The numbers in colored rectangles represent gene numbers in the module. The color scale bar on the right shows the correlation range from negative to positive

**Fig. 5:**
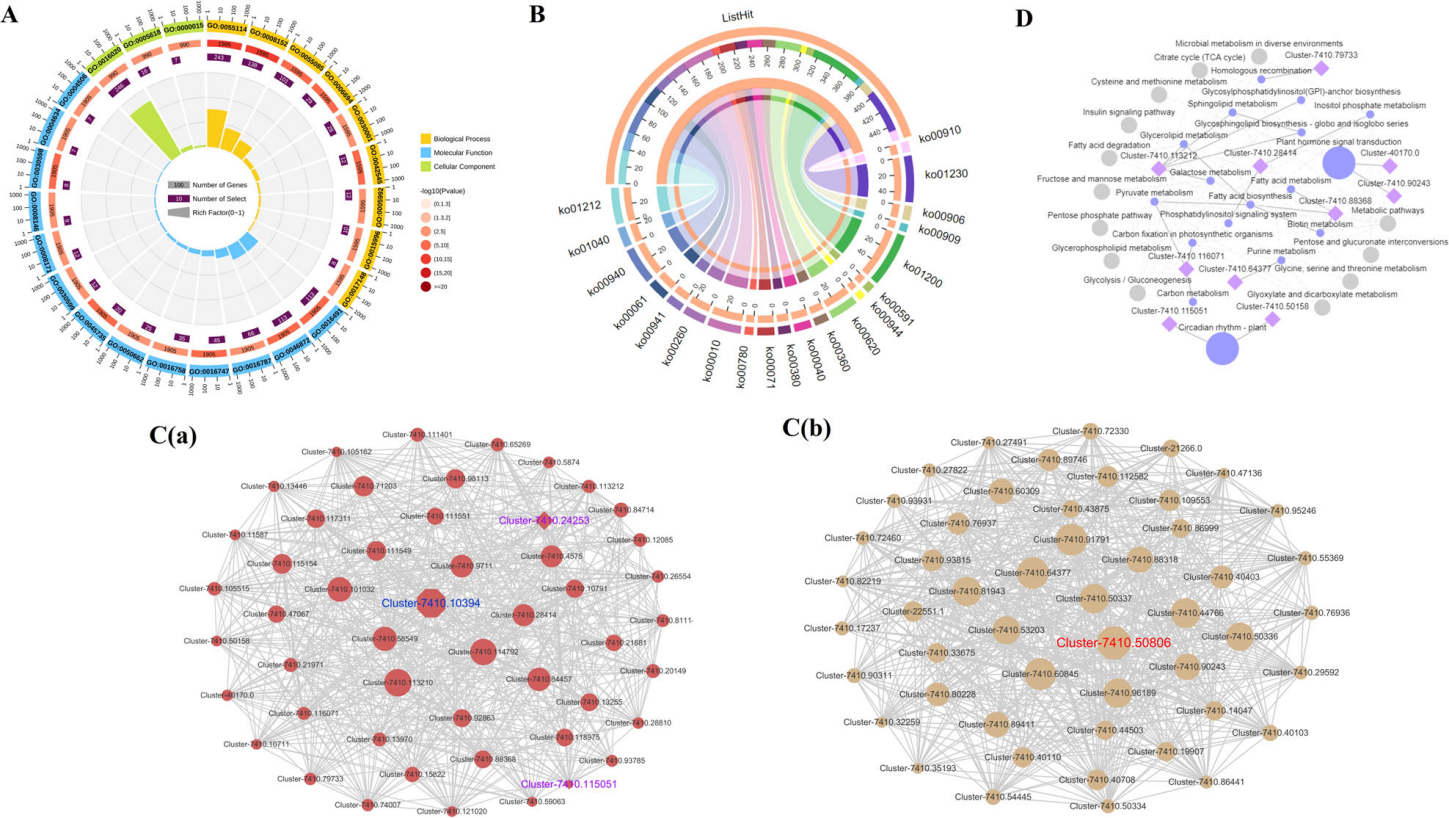
Enrichment analysis and gene networks of WGCNA modules in developing *C. oleifera* seeds. **A** GO circle plot displaying gene annotation enrichment analysis. **B** The top 20 KEGG pathway enrichment categories of these genes. Detailed information is listed in Table S8. **C** Cytoscape represents the top 50 coexpressed genes in the “indianred” (a) and “tan2” (b) modules. **D** KEGG pathway enrichment analysis of the hub genes

To identify the WGCNA modules related to oil quality during seed ripening of *C. oleifera*, a coexpression network was constructed by combining dramatic changes in total flavonoids, oil, and major fatty acids with high-throughput RNA-seq datasets. A total of 92 distinct modules consisting of 25,955 nonredundant unigenes were labeled in different colors and presented in the form of a cluster dendrogram, network heatmap, and trait heatmap, where the gray module represented genes that were not assigned to any specific module and had no reference significance (Fig. 4 and Table S8). Remarkably, two unique modules containing 4350 unigenes were highly correlated with the accumulation patterns of total flavonoids, oil, and fatty acids, where the absolute correlation coefficients were greater than 0.8 (*p* value ≤ 0.01; indianred and tan2). We then depicted the heatmaps and bar plots of genes across all samples to specifically detect the transcriptional expression profiles of these modules, among which the eigengene expression in the indianred module was the highest at the S1 stage (mean = 0.35). In contrast, the eigengene of the tan2 module showed higher expression at the S4 stage (mean = 0.34) than at the other stages (Fig. S1 and Table S8). Subsequent enrichment analyses were performed to explore the biological functions underlying the transcriptome in the above modules. As shown in Fig. 5A and Table S8, the significantly enriched GO terms were responsible for metabolic process and oxidation–reduction process. In addition, the KEGG pathways participated mainly in fatty acid metabolism, biosynthesis of UFAs, phenylpropanoid biosynthesis, fatty acid biosynthesis, and flavonoid biosynthesis, a result that was in accordance with the previous results of this study (Fig. 5B and Table S8). We noted that 92 out of the 4350 unigenes encoded 39 key enzymes, such as lipoxygenase (LOX1_5), acyl-CoA oxidase (ACOX), aldehyde dehydrogenase (ALDH), ACAT, CAD, HCT, and 4CL, and these unigenes were also found in the preceding analysis.

Furthermore, based on the eigengene connectivity (KME) values, the top 50 genes in the indianred and tan2 modules were selected separately to generate the coexpression subnetworks visualized using Cytoscape_v.3.7.1 to search for putative candidates with important contributions (Fig. 5C). Details of the coexpressed genes in the subnetworks are listed in Table S8. The highlighted gene (Cluster-7410.10394) encoding protein trichome birefringence had the highest KME value and was most closely associated with other node genes in the indianred module; similarly, the gene (Cluster-7410.50806) encoding retrovirus-related Pol polyprotein belonged to the core member of the tan2 module. Moreover, KEGG classification analysis of these unigenes provided additional information concerning the enriched biological pathways, including circadian rhythm-plant, glycosphingolipid, and biosynthesis-globo series (Fig. 5D and Table S8). Finally, we used the 12 algorithms of Cytohubba to screen out more crucial hub genes; 19 unigenes comprising 17 structural genes and two TFs consisting MADS intervening keratin-like and C-terminal (MIKC_MADS) and type-B authentic response regulator (ARR-B) coding genes were of potential research significance.

#### Identification and functional annotation of DEGs related to flavonoid and oil anabolism

There was a highly significant negative correlation (−0.8728) between the total flavonoid content and oil content in *C. oleifera* seeds, and the expression levels of 1110 unigenes were remarkably correlated with the variation in the above two physiological indexes (*p* value < 0.05). These unigenes were mainly enriched in GO terms associated with membrane, metabolic process, and transmembrane transport. The proposed unigenes were further classified by KEGG analysis, among which several pathways, such as “glyoxylate and dicarboxylate metabolism”, “flavonoid biosynthesis”, and “photosynthesis-antenna proteins”, were considerably modulated (Fig. S9 and Table S9). In addition, a PPI network of 161 unigenes (135 unique proteins) with confidence scores > 0.7 was constructed; the network included 65 unigenes (45 unique proteins) previously identified based on known metabolic processes such as phenylalanine ammonia-lyase (PAL), flavanone 3β-hydroxylase (F3H), and ACACA, while 96 unigenes (90 unique proteins) were newly found according to interrelation analysis (Fig. S9 and Table S9). These interacting proteins could be divided into six groups. In Cluster 1, a total of 34 closely linked proteins participated in carbon metabolism and glycolysis/gluconeogenesis. Cluster 2 covered 26 proteins that played crucial roles in arginine and proline metabolism. Cluster 3 comprised 25 proteins with different functions, including phenylpropanoid biosynthesis and phenylalanine metabolism. There were 21 proteins in Cluster 4, and these proteins were important for fatty acid metabolism, starch and sucrose metabolism, and fatty acid biosynthesis. Moreover, 19 proteins were assigned to Cluster 5, most of which were related to glyoxylate and dicarboxylate metabolism. Finally, ten proteins engaged in flavonoid biosynthesis were gathered in Cluster 6. Eight unigenes listed in this interacting network were also speculated to be key factors involved in the regulation of flavonoid and oil anabolism. Arogenate dehydratase (ADT, Cluster-7410.66059) and aspartate aminotransferase (AAT, Cluster-7410.42278) interacted directly with PAL. Auxin response factor ARF, Cluster-7410.77325 could interact with F3H. There was an obvious interaction between cysteine protease (Cluster-7410.66445) and peroxidase as well as a close relationship between beta-fructofuranosidase (INV, Cluster-21049.0) and beta-glucosidase (bglB).

### Proteomics analysis

#### General information for protein identification

In this study, the uniform distribution and high repeatability of bands on an SDS-PAGE gel indicated that the quality of extracted proteins was suitable for subsequent analyses (Fig. S10). A total of 1,337,264 spectra were generated, of which 134,352 were effective after the removal of the low-scoring spectra. By searching the Mascot engine, 27,674 unique peptides were inferred, and 5541 proteins were confidently identified. Among these, 4516 proteins were obtained from at least two experiments, and 3619 proteins were expressed over all three trials. In terms of protein mass distribution, we found good coverage for the molecular weight, ranging from 10 to 200 kDa (Table S10). These recognized proteins were acquired from 126 plant species by searching against the NR database (Fig. S10 and Table S10). The largest portion comprised 1508 proteins with strong sequence homology to *Actinidia chinensis* var. *chinensis*, followed by 113 proteins related to *Camellia sinensis*. As shown in Fig. S10 and Table S10, 630 proteins were of unknown functions or lacked KOG annotation information, and the remaining 2052 proteins were divided into 24 groups. The main functional categories were posttranslational modification, protein turnover, chaperones (O, 15.26%), and general function prediction only (R, 10.35%).

#### Quantitative comparison of protein expression during seed development

**Fig. 6:**
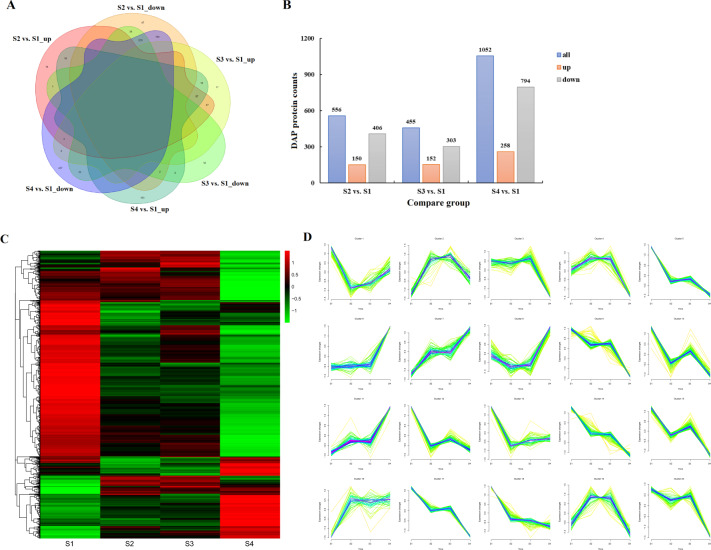
Expression analysis and quantitative comparison of the recognized DAPs in developing *C. oleifera* seeds. **A** Venn diagram of the shared and unique DAPs among three pairwise comparisons (S2 vs. S1, S3 vs. S1, S4 vs. S1, S1 as the control). The overlapping regions indicate the number of shared proteins. **B** Histogram showing the number of up- and downregulated DAPs in each compared group. **C** Hierarchical cluster heatmap of the recognized DAPs in four development periods. The colored bars indicate the changes in protein abundance after normalization; similar colors displayed by DAPs represent high correlation coefficients. The green color represents a low expression level, and the red color represents a high expression level. **D** Space-time clustering analysis of the recognized DAPs in developing *C. oleifera* seeds

Applying the cutoff threshold of a 1.2-fold change for differential accumulation together with the number of unique peptides ≥ 1, 1228 DAPs were recognized (Fig. 6A, B and Table S11). Compared with the S1 stage, 556 DAPs were identified at the S2 stage, among which 150 proteins displayed an increase in abundance; the S3 vs. S1 comparison group contained 455 DAPs consisting of 152 upregulated and 303 downregulated proteins; in the S4 vs. S1 comparison, 258 upregulated and 794 downregulated protein species were detected. Notably, 287 DAPs were shared in three pairwise comparisons; nearly all of these DAPs exhibited the same change trend, with only three proteins changing the direction of their expression. In addition, 61, 29, and 590 DAPs were found to be specific to the S2 vs. S1, S3 vs. S1, and S4 vs. S1 pairs, respectively.

As illustrated in Fig. 6C, D, hierarchical clustering and timing analysis revealed that the protein expression profiles in the S2 and S3 stages were closer to each other. According to their relative abundances, the DAPs were further assigned to 20 clusters. Stage-specific patterns were present in almost all the clusters, although in general, protein levels were only moderately changed. The largest category, Cluster 9, comprised 91 proteins showing a marked drop at prophase, then a slight increase from the S2 to S3 stages, and again a dramatic decline at the S4 stage. Cluster 2 referred to 41 proteins that demonstrated considerable accumulation in abundance up to a Z-score of approximately 1.0 and then decreased rapidly before reaching a peak at the S3 stage. Cluster 4 consisted of 59 proteins exhibiting an intermediate initial expression level that sharply increased to a maximum at the S2 stage and then remained fairly constant until the S3 stage. Sixty members were grouped into Cluster 6, in which proteins displayed a gradual increase in expression over the entire course of seed ripening. This trend was opposite that in Cluster 14.

#### Overall GO and KEGG pathway enrichment analysis

**Fig. 7:**
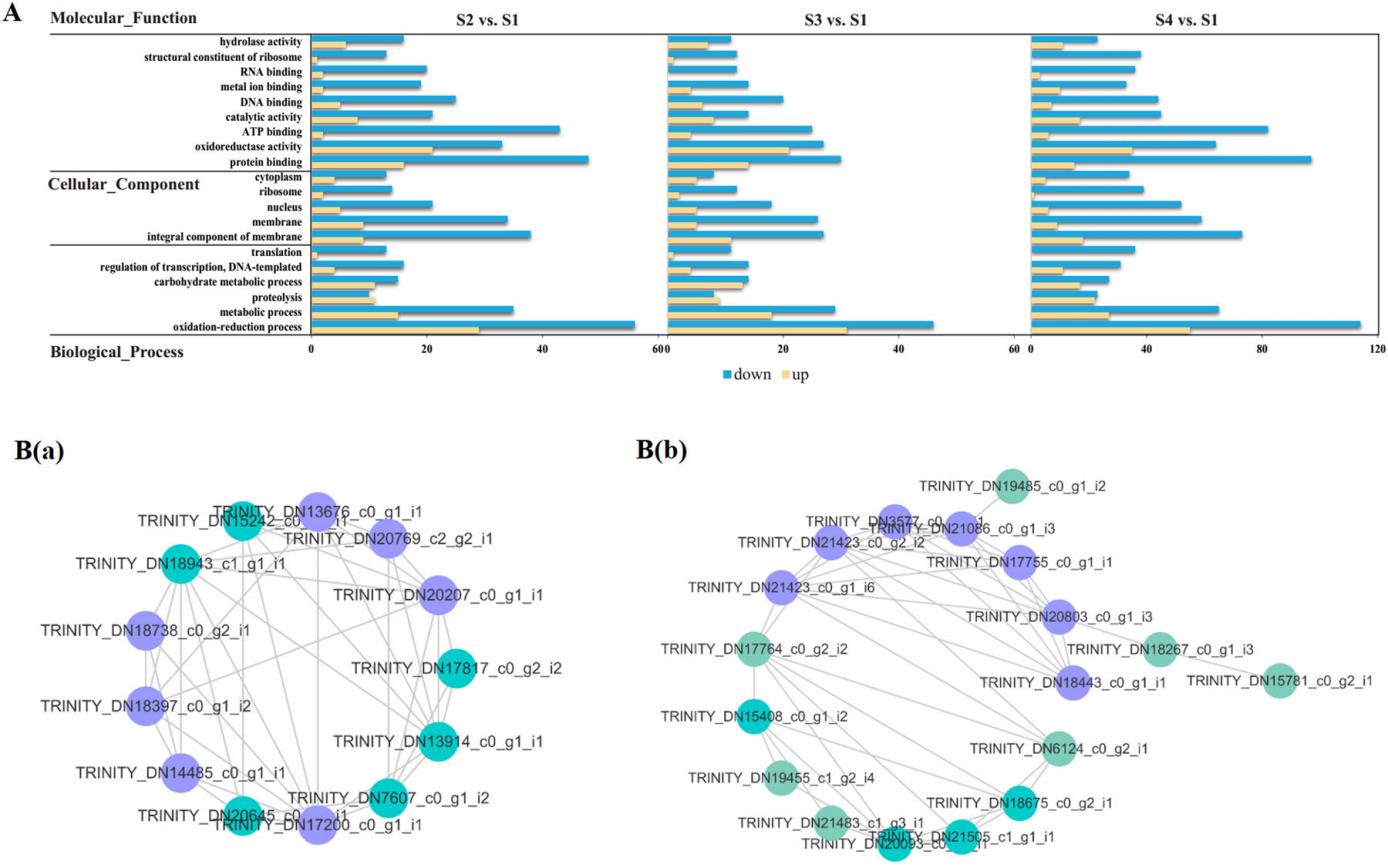
GO-based functional classification and protein–protein interactions of the recognized DAPs in developing *C. oleifera* seeds. **A** Top 20 GO categories for the recognized DAPs in the proteome. Yellow and blue bars represent up- and downregulated proteins in three main GO domains, respectively. **B** Interaction networks among the unique proteins involved in flavonoid biosynthesis (a) and fatty acid metabolism (b) pathways. The network nodes represent proteins, and the edges represent predicted functional associations between two proteins. Detailed information on protein names and abbreviations is found in Supplementary Table S13

GO enrichment analysis was used to clarify the functional distributions of proteins during the hull development of *C. oleifera* seeds. A bar graph of GO classifications is presented in Fig. S11 and Table S12. A total of 15,395 proteins corresponding to three major subsets were obtained, among which the categories of binding and cellular process were dominant. Furthermore, the most abundant GO terms of DAPs were screened for visualization (Fig. 7A). The results showed that the represented subclasses were oxidation–reduction process of the biological process category, integral component of the membrane for the cellular component category, and protein binding for the molecular function category. It was noteworthy that the S3 vs. S1 pair had the lowest number of DAPs mapped with GO information, which was inconsistent with the transcriptomic data.

The DAPs were coordinated with each other in vivo to express their biological functions, suggesting that our understanding of important metabolic processes during seed ripening could be further broadened by pathway-based annotation. According to the results, all identified proteins were categorized into 29 classes, mainly involving translation and carbohydrate metabolism (Fig. S12 and Table S12). A bubble chart of the top 20 KEGG pathways with *p* values less than 0.01 is plotted in Fig. S12. The universally enriched pathways shared among the three pairwise comparisons included fatty acid degradation, phenylpropanoid biosynthesis, and fatty acid metabolism. Specifically, the DAPs involved in α-linolenic acid metabolism were quite active in both the S3 vs. S1 and S4 vs. S1 comparison groups.

#### Identification and interaction network analysis of flavonoid and fatty acid anabolism-related DAPs, including TFs

As shown in Table S12, six DAPs took part in the flavonoid biosynthesis process, including F3Hs, DFR, trans-cinnamate 4-monooxygenase (C4H), flavonol synthase (FLS), and chalcone synthase (CHS). Four proteins were significantly downregulated in the three comparison groups. Some key enzymes for phenylpropanoid biosynthesis were detected among the 22 DAPs that comprised caffeic acid 3-O-methyltransferase (COMT), 4CL, and 20 other proteins. In particular, 13 members of the WRKY, bHLH, bZIP, MYB-related, and tryptophan-aspartate (WD40) repeat protein families could regulate flavonoid synthesis; of these, one bHLH was markedly upregulated in three pairwise comparisons, whereas one MYB-related (TRINITY_DN19768_c2_g2_i2) and two WD40 proteins were downregulated. Moreover, there were 18 DAPs related to fatty acid biosynthesis, including [acyl-carrier-protein] desaturases (FAB2s), fatty acyl-ACP thioesterase A (FATA), and 13 other proteins. Palmitoyl-protein thioesterase and 17β-estradiol 17-dehydrogenase (KAR) were associated with fatty acid elongation. A total of 14 DAPs composed of enoyl-CoA hydratases (MFP2s), ADHs, and 10 others were detected in the fatty acid degradation process. Moreover, 15 DAPs were identified in the α-linolenic acid metabolism pathway, and LOX1_5 was a downregulated protein (lowest at the S1 stage) that participated in linoleic acid metabolism. Analysis of the iTRAQ-based data also revealed eight DAPs that were annotated as TFs involved in fatty acid metabolism, and the proteins belonging to the AP2 and HD-ZIP families were all downregulated, a result that was not in accordance with the transcriptome results.

To elucidate the possible relationships among the protein species related to seed maturation of *C. oleifera*, PPI networks were generated based on the data from *Arabidopsis thaliana*. Given the potential size of the visualization image, the interacting proteins of particular interest were further extracted from the whole network, and two complex subnetworks were constructed (Fig. 7B). Specifically, 22 DAPs were associated with flavonoid biosynthesis, among which 13 DAPs (11 unique) represented a strongly interactive network. Nodes in different colors belonged to two major modules. Seven proteins (all unique) were assigned to Cluster 1, and the seed protein was coniferyl-ALDH (REF, TRINITY_DN20769_c2_g2_i1). These proteins mainly participate in carbohydrate transport and metabolism. Cluster 2 was composed of five enzymes, with the core protein being peroxidase (TRINITY_DN17817_c0_g2_i2), and this group included six proteins functioning in secondary metabolite biosynthesis, transport, and catabolism. In addition, 40 DAPs involved in fatty acid metabolic processes constituted an interaction network that contained 20 nodes and 45 edges. Eleven DAPs (eight unique) were divided into two functional modules forming tightly connected clusters. Seven proteins (four unique) were defined as Cluster 1, which was organized around ACOX (TRINITY_DN17755_c0_g1_i1). These proteins are known enzymes that participate in lipid transport and metabolism as well as amino acid transport and metabolism. Four proteins (all unique) were linked with Cluster 2, wherein FATA (TRINITY_DN15408_c0_g1_i2) was deemed to be the central protein. Taken together, this information provided some preliminary insights into the relationship networks concerning flavonoid and fatty acid anabolism. However, the above results were not perfectly consistent with those predicted by the transcriptome and therefore need to be verified by yeast two-hybrid experiments in the future.

#### Analysis of the protein coexpression network

**Fig. 8:**
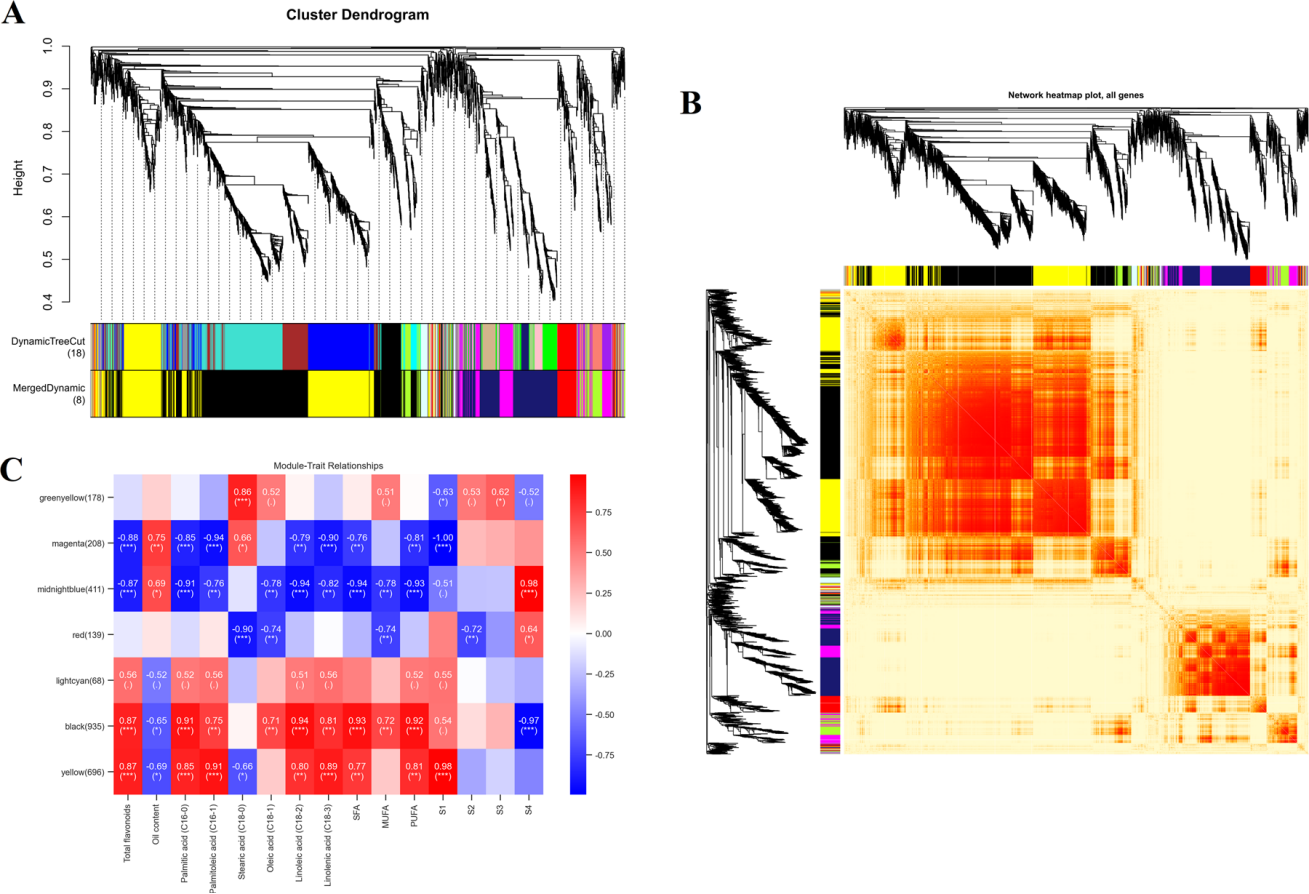
Weighted gene coexpression network analysis (WGCNA) of the identified proteins in developing *C. oleifera* seeds. **A** Protein dendrogram obtained by clustering the dissimilarity based on consensus topological overlap, with each tree branch constituting a module and each leaf representing one protein. Each colored row indicates a color-coded module that contains a group of highly interconnected proteins. **B** Heatmap plot of the topological overlap in the protein network. Darker squares along the diagonal correspond to modules. **C** Module eigengene physiological indexes and sample correlations. The numbers in colored rectangles represent protein numbers in the module. The color scale bar on the right shows the correlation range from negative to positive

**Fig. 9:**
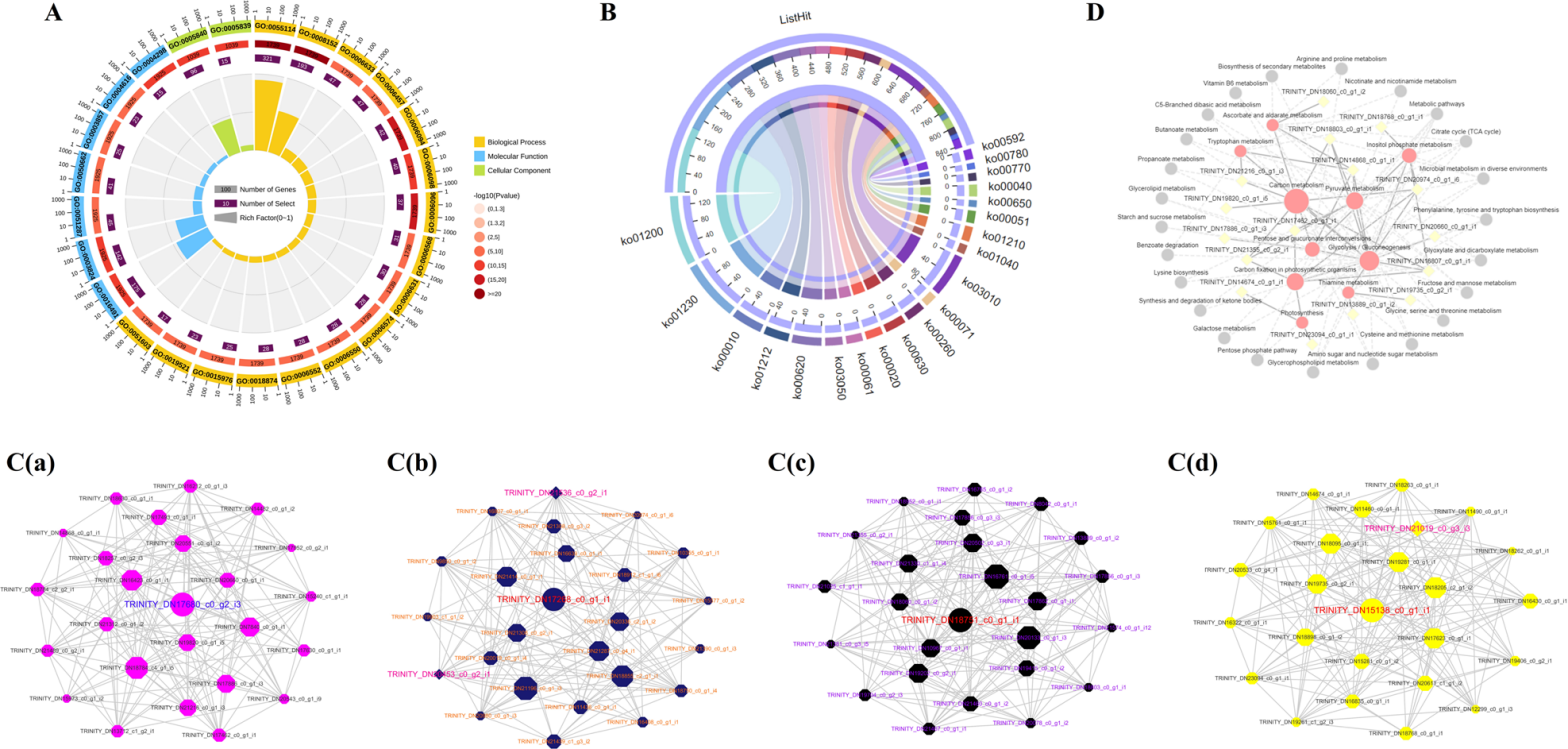
Enrichment analysis and protein networks of WGCNA modules in developing *C. oleifera* seeds. **A** GO circle plot displaying protein annotation enrichment analysis. **B** The top 20 KEGG pathway enrichment categories of these proteins. Detailed information is listed in Table S14. **C** Cytoscape represents the top 50 coexpressed proteins in the “indianred” (a) and “tan2” (b) modules. **D** KEGG pathway enrichment analysis of the hub proteins

Through WGCNA, eight distinct modules were constructed based on the coexpression patterns of 2682 individual proteins (Fig. 8A, B and Table S14). The contents of total flavonoids, oil, and major fatty acids at each mature stage were used as phenotypic data for the analysis of module-trait correlations (Fig. 8C and Table S14). Of these coexpressed protein networks, four specific modules composed of 2250 genes had strong associations with flavonoids, oil, and fatty acids, with absolute correlation coefficients greater than 0.6 (*p* value ≤ 0.05; magenta, midnightblue, black, and yellow). Combined with their heatmaps (Fig. S14 and Table S14), we found that the eigengenes of the black and yellow modules showed the highest expression at the S1 stage (mean = 0.27 and mean = 0.49), whereas the eigengene expression of the magenta and midnightblue modules exhibited the lowest levels at the S1 stage. The significantly overrepresented GO categories were further examined in the WGCNA modules mentioned above, whose proteins were predominantly enriched in oxidation-reduction process and metabolic process (Fig. 9A and Table S14). In addition, these unique proteins were mapped to 51 KEGG pathways, including carbon metabolism, biosynthesis of amino acids, glycolysis/gluconeogenesis, and fatty acid metabolism (Fig. 9B). Coincidentally, 62 critical proteins corresponding to 34 important enzymes (e.g., peroxidase, CAD, and FAB2) were also identified in previous research results.

Similar to those found in the transcriptome analysis, the top 25 proteins in the magenta, midnightblue, black, and yellow modules were selected according to their KME values to produce four coexpression subnetworks (Fig. 9C and Table S14). The proposed proteins are listed with their annotations in Table S14, among which succinate-semialdehyde dehydrogenase (TRINITY_DN17680_c0_g2_i3), GEM-like protein (TRINITY_DN17268_c0_g1_i1), RuBisCO large subunit-binding protein subunit alpha (TRINITY_DN18751_c0_g1_i1), and an uncharacterized protein (TRINITY_DN15138_c0_g1_i1) were considered the respective centers in the magenta, midnightblue, black, and yellow modules. In addition, these proteins were assigned to biochemical pathways in the KEGG database, mainly glycolysis/gluconeogenesis and thiamine metabolism (Fig. 9D, Table S14). Most notably, the hub proteins screened by Cytohubba’s 12 algorithms contained three TFs. B3 (TRINITY_DN21536_c0_g2_i1) and bHLH (TRINITY_DN20453_c0_g2_i1), located on the periphery of the midnightblue subnetwork, were basically upregulated throughout the whole ripening period of *C. oleifera* seeds, while the abundance of AP2 (TRINITY_DN21019_c0_g3_i3), situated in the outer ring of the yellow subnetwork, was lower in the S4 stage than in other phases, suggesting possible roles in regulating the accumulation of flavonoids, oil, and fatty acids.

#### Identification and functional analysis of DAPs related to flavonoid and oil anabolism

The contents of total flavonoids and oil in *C. oleifera* seeds showed obvious correlations with the abundance of 177 proteins (*p* value < 0.05). Specifically, these proteins corresponded to 386 GO terms. The oxidation-reduction process and cellular aromatic compound metabolic process were considerably enriched. KEGG analysis indicated that the proteins could be mapped to 74 pathways, of which 11 were significantly enriched, mainly involving carbon metabolism and the biosynthesis of amino acids (Fig. S15 and Table S15). Furthermore, a total of 65 unique proteins identified in this research were annotated in STRING and used to construct the PPI network (Fig. S15 and Table S15). Intriguingly, 14 unique proteins (e.g., CHS, F3H, FLS, and 4CL) were recognized based on their related metabolic processes, whereas 51 unique proteins were discovered by the association study. Finally, these protein species were presented in six main groups. Cluster 1 contained 18 proteins involved in the TCA cycle, galactose metabolism, and peroxisomes in the endoplasmic reticulum. Cluster 2 was composed of 13 proteins related to phenylpropanoid biosynthesis, flavonoid biosynthesis, and phenylalanine metabolism. Moreover, 11 proteins were gathered in Cluster 3; these proteins primarily belonged to 2-oxocarboxylic acid metabolism and carbon metabolism. Similarly, ten proteins that participated in the biosynthesis of amino acids were assigned to Cluster 4. Cluster 5 consisted of ten ribosome-associated proteins. In Cluster 6, three unique proteins were observed, namely, AT1G20580, NRPB11, and NRPD2A. Notably, eight proteins present in this interactive network were also conjectured to be the key factors affecting the dynamic changes of total flavonoids and oil contents in *C. oleifera* seeds, among which CHI-like protein (CHIL, TRINITY_DN21343_c1_g1_i2) interacted strongly with CHI, F3H, and FLS. Likewise, glycosyltransferase (GT, TRINITY_DN19890_c0_g2_i1) was linked with F3H. There was an obvious interaction between NAD(P)-binding Rossmann-fold superfamily protein (TRINITY_DN18784_c2_g2_i1) and CAD. In addition, ATP-dependent (S)-NAD(P)H-hydrate dehydratase (TRINITY_DN20543_c0_g1_i9), tubulin beta-6 chain (TRINITY_DN21381_c0_g3_i5), melibiase (TRINITY_DN19655_c0_g1_i4), glycosyl hydrolase (TRINITY_DN19932_c1_g1_i4), and peroxiredoxin (PRDX2F, TRINITY_DN17999_c0_g1_i1) showed clear interactions with PRDX6.

#### Conjoint analysis of transcriptome and proteome data

To evaluate the congruence between the transcriptome and proteome, as well as to understand how transcribed mRNA was manifested at the protein level, we conducted a global combination analysis of RNA-seq and iTRAQ assays (Fig. S16 and Table S16). A total of 2660 proteins could be matched to unigenes; however, most proteins and their transcripts did not meet the requirement for discrepancy accumulation. Approximately 98% of the DAPs were covered by RNA sequencing profiles. For the comparison of S2 vs. S1, the expression tendencies of 21 DAPs (14 upregulated and 7 downregulated proteins) agreed with the transcriptome data. Compared with stage S1, 140 DEGs showed the correlated regulation of both transcription and translation levels at stage S3. In the S4 vs. S1 pair, 223 DAPs overlapped with the transcriptomic results, among which 52 members exhibited an opposite changing trend across the two levels.

In addition, we focused on the overlap between DEGs and DAPs that shared the same regulatory status across the three comparisons. There were only four upregulated members (lowest at the S1 stage), basic 7S globulin (7SB1), malate synthase (MASY), late embryogenesis abundant protein (LEA14), and one annotated as coding for an uncharacterized protein. The three downregulated members were vinorine synthase (VINSY), glutelin (GLUA2), and carboxylesterase (CXE12). This phenomenon demonstrated that time-dependent delays or regulatory processes occurring from transcript to protein levels might directly affect protein synthesis. We next conducted an association analysis using the quantitative data of unigenes and proteins. The abundance levels of the protein species and their corresponding mRNAs appeared to have lower correlation values; the Spearman correlation coefficients for the three comparisons were 0.13, 0.05, and 0.16 (Fig. S17 and Table S16). Furthermore, poor correlations were also observed between DEGs and DAPs, with coefficients of 0.09–0.28. Significantly, the S4 vs. S1 pair had the largest number of mutually relevant members, and similar results were found when their expression patterns were compared via heatmaps (Fig. S18).

### Bioinformatic analysis of the matched DEGs and DAPs

**Fig. 10:**
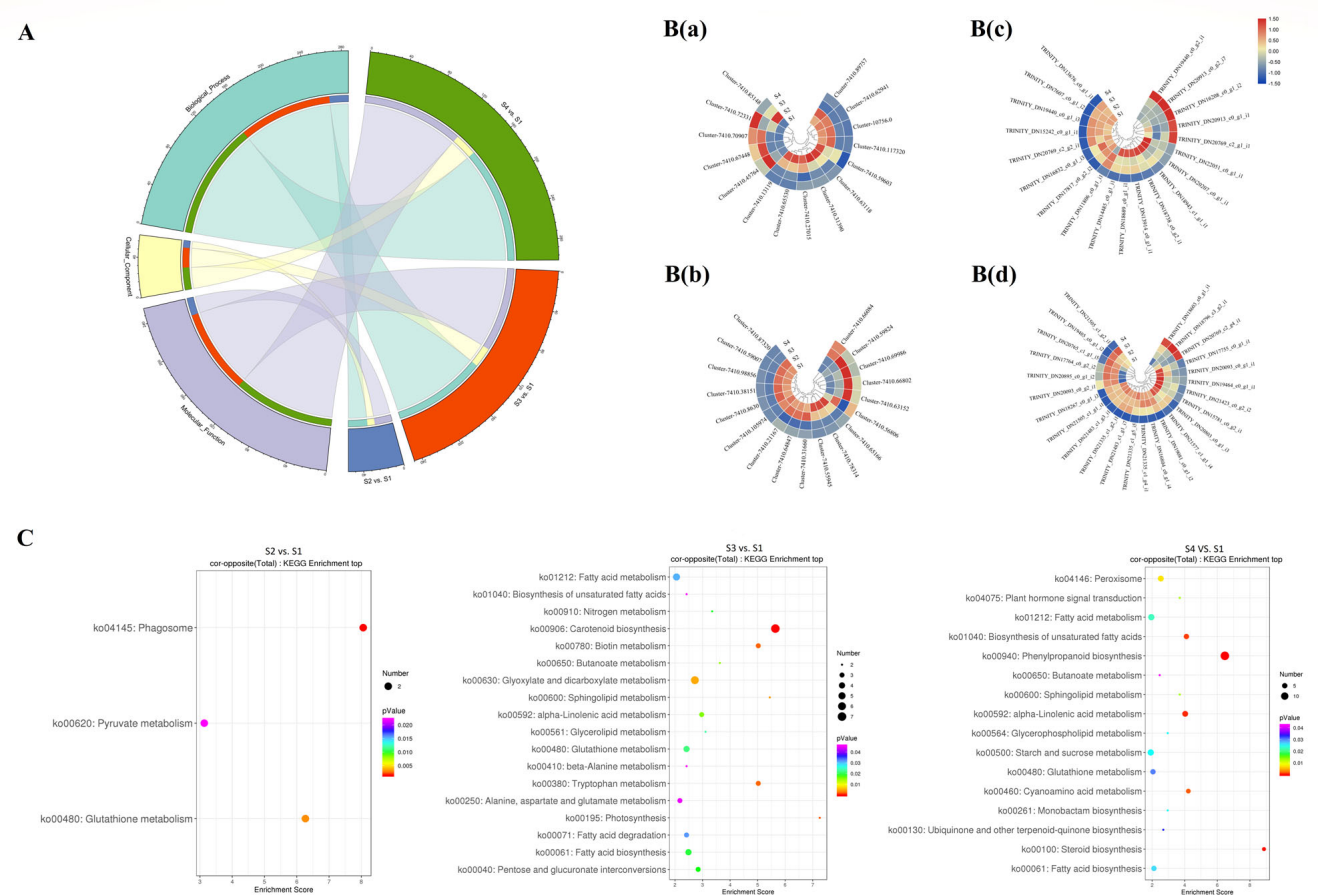
Enrichment analysis and hierarchical cluster heatmap of the coexpressed DEGs and DAPs. **A** GO analysis of the cognate DEGs and DAPs in the three pairwise comparisons with the smallest *p* values (< 0.05) and no fewer than two members. **B** Abundance patterns of unigenes related to flavonoid biosynthesis (a) and fatty acid metabolism (b) pathways. Abundance patterns of the proteins related to flavonoid biosynthesis (c) and fatty acid metabolism (d) pathways. *Z*-score fold change values are shown on a color scale that is proportional to the abundance of each member. **C** KEGG pathway enrichment of the three comparative analyses. The rich factor is the percentage of members out of the total number detected. The bubble size represents the number of members detected in the KEGG pathway, and the color of the bubble represents the *p* value

We further performed GO distribution analysis of these members, and the enriched outputs of their biological processes, molecular functions, and cellular components were presented (Fig. 10A and Table S17). The S4 vs. S1 group had the most abundant DEGs or DAPs, followed by the S3 vs. S1 pair. Regarding all three comparisons, oxidation-reduction process and metabolic process occupied the largest proportion, and the integral component of the membrane and oxidoreductase activity were the main categories in both the S3 vs. S1 and S4 vs. S1 pairwise comparisons. We found that seven GO terms were significantly enriched in the S2 vs. S1 pair, among which the abundance changes of DAPs related to transferase activity agreed with the transcription levels. The proportions of three GO classifications with the two omics datasets were compared and illustrated by a double pie chart (Fig. S19).

The regulated genes (differentially expressed at both the mRNA and protein levels) were classified into different KEGG pathways, where the screening criterion was a *p* value less than 0.05 (Fig. 10B and Table S17). The phagosome and glutathione metabolism were significantly modulated in the S2 vs. S1 comparison. With regard to the S3 vs. S1 group, the members were mainly involved in carotenoid biosynthesis as well as glyoxylate and dicarboxylate metabolism. For the S4 vs. S1 pair, we discovered that the most universally enriched pathways were linked to metabolic processes such as fatty acid metabolism and α-linolenic acid metabolism. The expression abundances of DEGs and DAPs associated with fatty acid metabolism and fatty acid biosynthesis were all downregulated.

### DEGs and DAPs involved in flavonoid biosynthesis and fatty acid metabolism

Classification and annotation of the transcripts and protein species related to the “flavonoid biosynthesis” and “phenylpropanoid biosynthesis” pathways were completed to characterize their functions more comprehensively (Table S17). The results indicated that 20 genes or proteins were differentially regulated, among which six members corresponded to peroxidases. Three members belonged to CADs and were all downregulated in the S4 vs. S1 group. Two members were F3Hs, and one unigene (Cluster-7410.85148) therein was upregulated in the comparison of S2 vs. S1, while its corresponding protein was downregulated. Genes annotated as cinnamoyl-CoA reductase, F5H, 4CL, COMT, and FLS were downregulated at both the mRNA and protein levels in the S4 vs. S1 comparison. In addition, to better understand the expression profiles of candidate DEGs or DAPs during seed ripening, their abundances were estimated via hierarchical clustering (Fig. 10C). The unigene (Cluster-7410.72331) had the highest expression level at the S4 stage, in parallel with the corresponding two proteins (peroxidases). The changing trend of the unigene (Cluster-7410.45764) decreasing from S3 to S4 was opposite the pattern of the homologous protein (peroxidase). In the current work, 25 members were involved in several fatty acid metabolism-related pathways (Table S17). The expression tendencies of DEGs encoding FAB2 and hydroperoxide lyase agreed with their corresponding DAPs in the pairwise comparison of S4 vs. S1. In addition, three members were regarded as ACOX, wherein one unigene (Cluster-7410.78314) and its cognate protein were downregulated in both the S3 vs. S1 and S4 vs. S1 pairs. Moreover, the unigene (Cluster-7410.63152) was expressed at a higher level at the S3 stage than at the S1 stage, in contrast to its corresponding protein (ALDH). The abundances of the unigene (Cluster-7410.69986) and the unigene (Cluster-7410.66802) were lowest at the S1 stage, and their regulatory status contrasted with the proteins annotated as 12-oxophytodienoic acid reductase (OPR) and ACOX, respectively (Fig. 10C).

According to the known metabolic pathways combined with WGCNA and correlation analysis, five coexpressed transcripts and proteins (CADs, COMT, FLS, and 4CL) associated with oil quality during seed ripening of *C. oleifera* were screened out, among which one member of interest (FLS) was selected for the bioinformatics assay. The results indicated that this protein (TRINITY_DN18738_c0_g2_i1) was located in the cytosol and contained a specific domain of 2OG-FeII_Oxy that was conserved in the 2OG-Fe(II) oxygenase superfamily. Its amino acid sequence was highly homologous to the FLS sequences from other plants, including *Camellia fraterna* (98.53%, AUM57439.1), *C. sinensis* (99.41%, ARM53419.1), *Camellia nitidissima* (82.44%, ADZ28516.1), and *Nyssa sinensis* (79.70%, KAA8546373.1). In addition, a phylogenetic tree was constructed to further clarify the corresponding characteristics; the protein clustered with the known proteins of *C. sinensis* in a clade, revealing that they shared the most recent genetic relationship (Figs. S20 and S21).

### qRT-PCR validation of differential expression

**Fig. 11:**
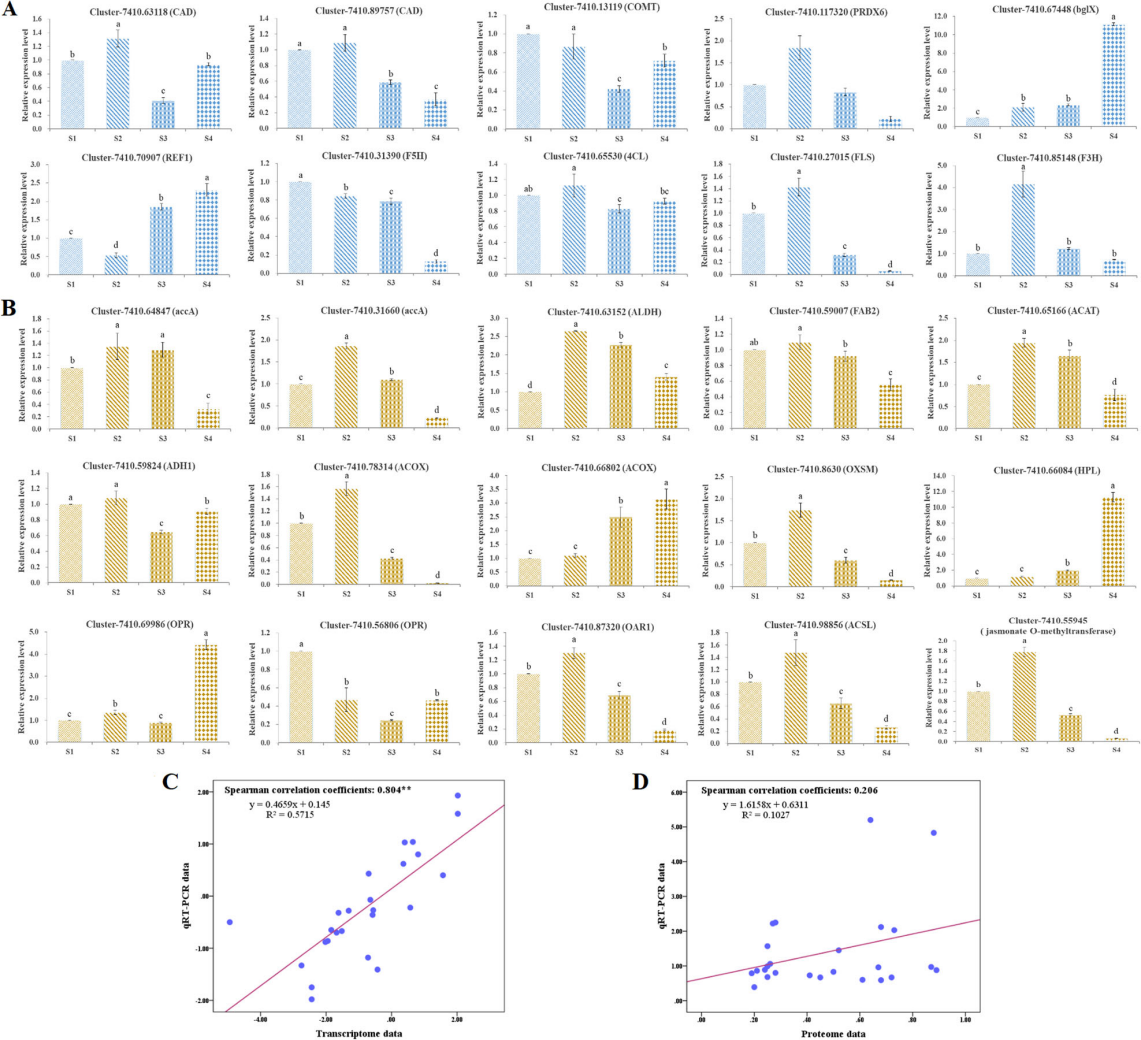
qRT-PCR verification of the expression profiles in developing *C. oleifera* seeds. The relative expression levels of candidate genes were calculated according to the 2^−ΔΔCt^ method using GAPDH as an internal reference gene. All data represent the mean values ± standard error of three biological replicates. Different letters above the columns indicate significant differences in seeds at four developmental phases based on one-way ANOVA (*p* < 0.05). The blue and yellow colors represent the genes associated with flavonoid biosynthesis (**A**) and fatty acid metabolism (**B**) pathways, respectively. Linear regression between the levels of qRT-PCR data and transcript expression (**C**) and protein accumulation (**D**)

To verify the reliability of the transcriptomic and proteomic data, 31 representative DEGs among the coexpressed mRNA and protein profiles potentially involved in flavonoid and fatty acid anabolism were selected for qRT-PCR assays (Fig. 11 and Table S18). The results were in general agreement with those from the RNA-seq, with a Spearman correlation coefficient of 0.804, indicating that the transcriptome data were able to reflect transcript abundance in this study. Nevertheless, the relationship between transcription and translation levels was not strong, suggesting that proteins had longer half-lives than mRNAs; the change trends of a few unigenes were similar to those of the proteins they encoded, implying that our iTRAQ results were basically accurate and credible.

## Discussion

### General features of the transcriptome and proteome

In recent years, the breeding objective for *C. oleifera* has gradually turned from high yield to high quality; however, several studies on accumulative regulation related to its fatty acids or other active ingredients are based solely on transcriptome sequencing, which cannot provide a complete biometabolic map. As a complementary analysis to transcriptomics, proteomics mainly delineates the protein expression profiles to characterize the functional aspects in living systems^32^. With that in mind, RNA-seq, iTRAQ, and GC-MS techniques were applied to preliminarily decipher the dynamic variation of nutritional components during seed maturation of *C. oleifera* from Hainan Island as well as their possible molecular mechanisms.

In our research, a total of 16,530 DEGs and 1228 DAPs were identified according to the stated thresholds, meaning that the data have enriched the current knowledge of the *C. oleifera* transcriptome and proteome. The largest number of DEGs was detected in the comparison of S3 vs. S1, while the most abundant DAPs were recognized in the S4 vs. S1 comparison, implying that greater changes in biological processes may appear in the mature phases. Although more than 98% of the differentially expressed proteins were covered by the transcriptomic results, poor concordance between the expression levels of DEGs and DAPs was observed, as reflected by the low Spearman correlation coefficients. This result was similar to those reported for potatoes^33^ and peppers^34^. A plausible explanation is that the fluctuation of transcription levels is more rapid than the changes in protein abundance, as the latter is accompanied by posttranscriptional modification, translational regulation, or the involvement of splicing events in cells^35,36^. Simultaneously, KEGG pathway analysis was performed to better interpret the complex metabolic networks related to the synthesis and degradation of flavonoids or fatty acids from the perspective of multiomics.

### Flavonoid biosynthesis

**Fig. 12:**
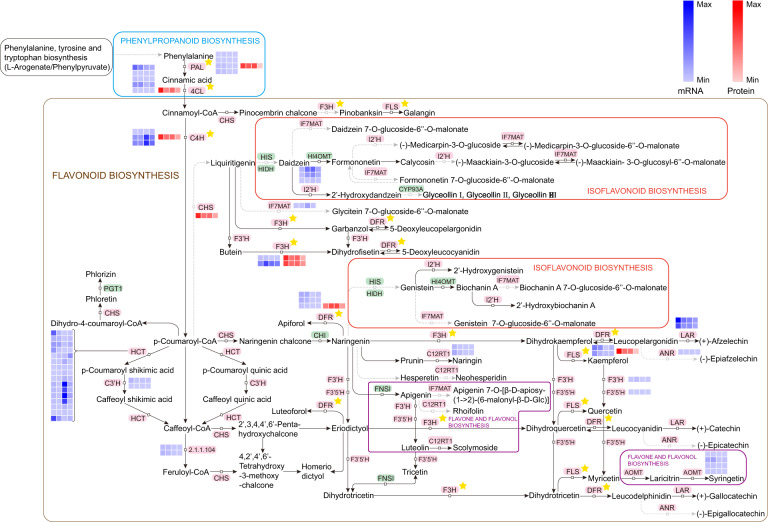
Visualization of protein and transcript expression in a biochemical pathway map related to flavonoid biosynthesis in developing *C. oleifera* seeds. The heatmap was plotted using fold change values from proteome data and log2 transformed gene expression values. Black characters with a pink background are enzymes. The asterisks represent the coexpression of encoded unigenes and proteins. *Z*-score fold change values are shown on a color scale that is proportional to the abundance of each unigene and protein

Flavonoids are a large group of polyphenolic secondary metabolites that are widespread in spermatophytic plants, and this group includes flavonols, flavones, flavan-3-ols, isoflavones, flavanones, and anthocyanidins^37^. There is increasing evidence that flavonoid components have medicinal properties such as antioxidant activity, anti-inflammatory activity, antitumor activity, vascular activity, estrogenic activity, and other biological functions^38^. Flavonoids are products of phenylpropanoid metabolism, which is considered to be a bridge connecting primary and secondary metabolism^39^. More concretely, this regulatory network begins from phenylalanine with *p*-coumaroyl-CoA acting as a precursor and is further channeled into the biosynthetic pathway of flavonoids through the catalysis of PAL and 4CL, which reside at critical positions for controlling the flow of carbon^40^. The expression levels of unigenes encoding these two gateway enzymes were downregulated continuously, and the abundances of their corresponding proteins also generally decreased along with seed ripening, in line with the changes in phenylpropanoid content. The involved enzymatic candidates annotated with protein and mRNA differential expression levels are shown in Fig. 12. Interestingly, multiple members could be annotated as the same enzyme, possibly because they belong to different alternative splicing transcripts as well as specific gene families^41^.

Three aromatic rings generated by CHS constitute the basic skeleton of all flavonoids^42^; only one such corresponding protein was identified in our dataset, and this displayed a similar regulation pattern that declined with increasing maturity. Chalcone is subsequently isomerized to naringenin (flavanone) by CHI, and dihydroflavonols are further formed with the participation of F3H^43^. The peak abundances of F3H proteins and their corresponding genes were basically in the S1 stage, whereas the CHIs were identified as having no differences during seed maturation. FLS is a committed enzyme that converts dihydrokaempferol, dihydroquercetin, and dihydromyricetin into aglycones (flavonols) by competing at crucial branch points with DFR^44^. Phylogenetic analysis showed that one FLS protein recognized in this work had the most recent genetic relationship with those of *C. sinensis* and *C. fraterna*, indicating that the FLS gene was relatively conserved. However, the abundances of FLS- and DFR-encoded proteins might not be consistently correlated with the expression levels of their cognate transcripts. The same situation was reported in a recent profiling study on leaves at different maturity levels in *C. sinensis* L^45^. This result was largely a consequence of the lag between mRNA appearance and protein synthesis^46^. Anthocyanidin reductase (ANR) and LAR are key downstream enzymes for the biosynthesis of non-epi- and epi-types of catechins, afzelechin, and gallocatechin^47^. Only one transcript annotated as ANR was found to be expressed at a low level, and the differences among four developmental phases were marginal. We also discovered two LAR genes that were expressed substantially higher at the S1 stage than at the S4 stage. In addition, HCT has been considered a reversible enzyme under which *p*-coumaroyl CoA can be committed to lignin production. Li et al.^48^ indicated that the inhibition of HCT expression could result in the accumulation of flavonoids. In this work, most structural DEGs encoding HCT were abundantly expressed at the S3 stage, which might be one of the factors leading to a decrease in the flavonoid content as the seeds mature. Coincidentally, a similar inference was drawn from the result that in HCT-silenced plants, the metabolic flux was reoriented to flavonoids through CHS activity^49^. To summarize, the overall tendency of these positive regulatory enzymes related to flavonoid-derived compound biosynthesis was attenuated with seed maturity at both the proteome and transcriptome levels, confirming our metabolic results.

TFs, as proteins dominating the spatial and temporal changes of genetic transcription, are involved in organism development. Studies have revealed that certain TFs could control flavonoid metabolism in various organs or growth phases of plants^50,51^. Of particular note, the ternary MBW complex composed of R2R3-MYB, bHLH, and WD40 proteins regulates flavonoid biosynthesis by activating some downstream genes encoding CHS, F3H, FLS, and DFR, leading to the formation of diverse branches^52^. Specifically, WD40 proteins are not considered to have catalytic capability but rather seem to be a docking platform for the regulation of flavonoid synthesis^53^. In our research, the expression analysis showed that a MYB gene (Cluster-7410.73237) and a bHLH protein (TRINITY_DN20453_c0_g2_i1) were significantly upregulated in three comparisons, and thus, the two members above might have crucial roles in activating the late flavonoid biosynthetic genes of *C. oleifera* seeds. This is possibly due to the lack of a published genome and the limitations of current technology, as we only found a few subtypes of these transcriptional regulators. Even so, our results could still yield preliminary transcriptomic and proteomic data support for the coregulatory effects of TFs on flavonoid biosynthesis.

### Fatty acid metabolism

**Fig. 13:**
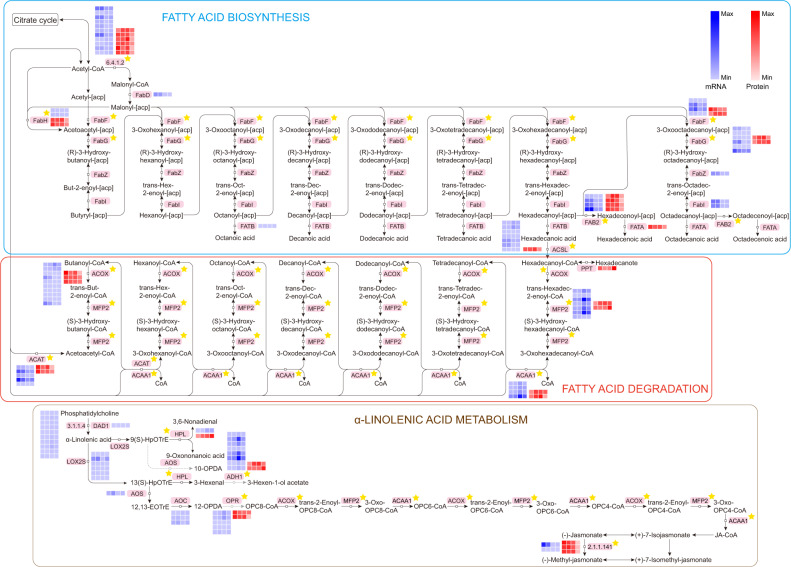
Visualization of protein and transcript expression in a biochemical pathway map related to fatty acid metabolism in developing *C. oleifera* seeds. The heatmap was plotted using fold change values from proteome data and log2 transformed gene expression values. Black characters with a pink background are enzymes. The asterisks represent the coexpression of encoded unigenes and proteins. *Z*-score fold change values are shown on a color scale that is proportional to the abundance of each unigene and protein

*C. oleifera* oil shares an extremely similar fatty acid profile with olive oil, and as such, it has been shown to be superior to soybean, castor, peanut, and sunflower oils. Therefore, it is listed among the priority healthy edible oils by the Food and Agriculture Organization^54^. Numerous studies have indicated that this oil is characterized by abundant UFAs that have significant health-related functions and therapeutic effects^55,56^. Because of this, systematic investigations and practical applications are expanding constantly. In the current work, a visible alteration in the fatty acid contents of oil samples was observed, as the contents increased rapidly at first, attaining a peak at the S2 stage, and then progressively declined. A similar phenomenon has been described in several oilseed species, i.e., a decrease in lipid content occurs at the very end of the seed maturation process^57,58^. To interpret this, one could consider that maturing seeds with low moisture content and no more trophic connections with plants must utilize part of their lipid reserves while completing oligosaccharide synthesis^59^. Intriguingly, we also found that there was a trade-off relationship between the changes in diverse components, implying possible conversions of palmitic acid to stearic acid, SFAs to UFAs, and PUFAs to MUFAs during the accumulation of fatty acids. Past studies have proven that substantial variation in fatty acid composition is regulated by certain key pathways^60,61^. However, information regarding the metabolic mechanisms underlying fatty acid biosynthesis in *C. oleifera* on Hainan Island is still limited. It needs to be stated that we have previously performed a comprehensive proteomic and transcriptomic analysis on the mature seeds of this plant by using a shotgun qualitative approach and RNA-seq technique (Illumina HiSeq X Ten platform) and preliminarily revealed the characteristics of fatty acids in seeds at the maturity stage^22^. On this basis, the present study further elucidated the dynamic changes in the functional protein profile and its mRNA transcriptional level during seed ripening of *C. oleifera* on Hainan Island via an iTRAQ-based quantitative method and RNA-seq technology (Illumina NovaSeq platform). It is hoped that the corresponding DEGs or DAPs discovered in our experiments will be useful for identifying some potential regulatory factors and providing molecular clues for profoundly studying fatty acid metabolism (Fig. 13).

Fatty acid biosynthesis is derived from acetyl-CoA with a chain length of C16 or C18 and mainly involves two enzyme systems: acetyl-CoA carboxylase (ACC) and fatty acid synthase complex^62^. This functional network is initially catalyzed by ACC and FabD to form malonyl-ACP, and then four committed steps are taken in turn, requiring the addition of two carbons, under the catalysis of 3-oxoacyl-[acyl-carrier-protein] synthases III (fabH, KAS III), 3-oxoacyl-[acyl-carrier-protein] reductase (fabG), FabZ, and enoyl-[acyl-carrier protein] reductase I (FabI). The product of the first synthetic cycle, butyryl-ACP, is the substrate for subsequent elongation rounds, each of which needs to use one molecule of malonyl-ACP and release carbon dioxide^9^. Furthermore, the condensation from C4 to C16 is carried out through 3-oxoacyl-(acyl-carrier protein) synthase I (FabB, KAS I) instead of fabH, while the reaction from C16 to C18 is conducted via 3-oxoacyl-[acyl-carrier-protein] synthase II (FabF, KAS II). Notably, acyl-CoA, as a lipid metabolic intermediate that participates in multiple physiological processes, is generated from free long-chain fatty acids catalyzed by ACSL^63^. In our study, these coexpressed transcripts and proteins showed basically similar change tendencies, first increasing and then declining with seed maturity, which was coincident with the fatty acid accumulation pattern. This means that the important enzymes mentioned above were positively related to the biosynthesis of fatty acids in a synergistic manner. Consequently, we speculated that the synthesis rate of fatty acids in the early period of seed maturation might be faster than that in the later period, whereas the consumption of free fatty acids was the opposite. A similar scenario was found in *Camellia chekiangoleosa*; that is, these fatty acid-synthesizing DEGs encoding ACC, FabF, FabG, and FabB were more highly expressed in the low-yield type than in the high-yield type, indicating a high level of fatty acid generation in the low-yield type. Thus, the low-yield type had a higher oil content than the high-yield type^64^.

For the degradation of fatty acids in mitochondria, ACAT can facilitate the condensation of two acetyl-CoAs to yield acetoacetyl-CoA, a common starting substrate for metabolite production^65^. We have previously demonstrated that this protein is the central one in the interaction network due to its strong relationships with many other proteins involved in lipid metabolic processes^22^. Two DAPs and five DEGs whose expression levels changed significantly during seed maturation were also identified in this work. MFP2 was the second multifunctional enzyme discovered in the peroxisomal β-oxidation pathway responsible for chain shortening of carboxylates^66^. The rapid accumulation of most MFP2 proteins and their corresponding mRNAs from the S1 to S4 stages agreed with the outputs of metabolism tests, indicating that the S4 stage possessed the lowest content of total fatty acids. This further illustrated that the enzyme could accelerate the decomposition rate of fatty acids with increasing maturity. Our results are in accordance with the findings in coix seed oil, suggesting that perhaps a similar regulatory mechanism is involved^67^. In addition, α-linolenic acid, which can attenuate a variety of inflammatory reactions, is typically consumed as part of a dietary supplement^68^. Reportedly, LOX as a major substrate takes part in the α-linolenic acid metabolic pathway and can catalyze linoleate oxidation in higher plants^69^. In the present study, we showed that six LOX-encoded genes were differentially expressed. ADH is capable of promoting the interconversion of alcohols to aldehydes or ketones, and the reaction requires NAD + or NADP + ^70^. Following the ripening of *C. oleifera* seeds, the DEGs encoding ADH were upregulated gradually at both the transcriptional and translational levels, peaking at the S3 stage, in agreement with the GC-MS data and in line with the prior research conclusion that the observed increases in ADHs and LOXs were correlated with a reduction in linoleic acid synthesis^67^. Conversely, Song et al.^71^ demonstrated that the ADH protein decreased markedly with advanced strawberry fruit maturity. This discrepancy may be due to genetic, environmental, or other factors that deserve to be thoroughly explored.

In recent years, TFs belonging to the AP2, B3, DOF, HD-ZIP, HAP3/CBP, and CHD3 families have been favored because of their ability to regulate fatty acid biosynthesis, thereby paving the way for increasing the yield and quality of vegetable oils. Instead of acting alone, they form a metabolic network. In our study, the proteins annotated as AP2 and HD-ZIP were all downregulated (lowest at the S1 stage). Ibáñez-Salazar et al.^72^ considered that the overexpression of DOF-type TF genes would increase lipid production in *Chlamydomonas reinhardtii* seeds. Unfortunately, although multiple differentially expressed DOF unigenes were detected, we could not find any of their homologous proteins in this experiment, possibly owing to accumulated transcripts not always being converted to cognate proteins. The investigation of TFs associated with fatty acid metabolism can provide a reference for varietal improvement in *C. oleifera*, but there are still some limitations: the structural characteristics of many TFs are unclear, and the functional mechanisms of downstream target genes remain to be clarified.

### Potential candidate genes and proteins detected by WGCNA and correlation analysis

Genes or proteins often participate in biological processes via coordinated expression^73^; hence, we employed the WGCNA method to construct coexpression networks separately and to identify several key modules associated with flavonoid, oil, and fatty acid metabolism in *C. oleifera* during seed ripening. The results of this study could also provide new insights into the corresponding molecular mechanisms. Regarding the transcriptome data, the indianred and tan2 modules were found to contain some high-degree hub genes that played critical roles in the network. Significantly, two TF coding genes with the same expression pattern of continuous decrease and then increase were screened out; MIKC_MADS (Cluster-7410.24253) belonging to the type II model is a member of the MADS-box TF superfamily whose genes are involved in virtually all aspects of plant development, especially in regulating biosynthesis of secondary metabolites in eukaryotes^74^. Meng et al.^75^ indicated that one MADS-box gene (GlMADS1) could control flavonoid production in *Ganoderma lucidum*. Li et al.^76^ reported that another MADS-box gene (EgMADS21) might modulate TAG assembly and PUFA accumulation in the maturation of oil palm fruit. ARR-B (Cluster-7410.115051) is characterized by a receiver domain followed by a DNA binding domain (GARP motif), thus acting as a TF^77^. The specific GARP TF family, which is distantly related to the MYB superfamily, contains genes with multiple plant functions^78^. Petridis et al.^79^ demonstrated that the GARP gene (At5g45580) was able to affect phenylpropanoid metabolism under low-temperature conditions, favoring the accumulation of flavonoids. It can be speculated that these two genes may also have similar functions. For proteome data, four distinct modules (magenta, midnightblue, black, and yellow) showed obvious correlations with the examined traits, and B3, bHLH, and AP2 proteins were found within the subnetworks built from the top proteins. This result is also entirely consistent with the prior analysis outcome of the present work. Considering this result, molecular biology research on these TF-related unigenes (proteins) recognized in our investigations should be carried out thoroughly in the future to fully understand the genetic regulators of flavonoid, oil, and fatty acid anabolism in *C. oleifera* seeds.

Remarkably, several research studies have confirmed that flavonoids have negative regulatory effects on fatty acid accumulation. Flavonoids can restrain fatty acid generation by competing for synthesizing substrates and inhibiting the expression of critical reductases FabG and FabI^80^. However, the absence of flavonoids may cause the enhancement of auxin, which makes plant seeds utilize more energy for carbon source transformation, finally leading to an increase in fatty acid content^81^. At the same time, our study also found that there was a significant negative correlation between the total flavonoid content and oil content with increasing maturity of *C. oleifera* seeds. Fortunately, five unigenes and eight proteins identified in this work were suggested to be the key factors involved in the regulation of flavonoid and oil anabolism. Among these, ADT encoded by the unigene (Cluster-7410.66059) is able to transform the prephenate produced from the shikimate-chorismate pathway into phenylalanine, which is not only the precursor of flavonoid biosynthesis but can also synthesize important neurotransmitters together with tyrosine, participating in fat metabolism^82,83^. In addition, AAT encoded by the unigene (Cluster-7410.42278) catalyzes the reversible transfer of the amino group of aspartate or glutamate to 2-oxoglutarate or oxaloacetate, which can then be converted to phosphoenolpyruvate associated with flavonoid biosynthesis, and acetyl-CoA related to fatty acid biosynthesis^84,85^. At the protein level, CHIL is a component of flavonoid metabolon that has been shown to physically interact with CHS of the same plant species by yeast two-hybrid and luciferase complementation imaging assays^86^. Moreover, this enzyme and fatty acid-binding protein (FAP) belong to different types of CHI polygene family members, and FAP can affect the biosynthesis of fatty acids in plant cells and their storage in developing embryos^87^. GT (TRINITY_DN19890_c0_g2_i1) can have an impact on the synthesis and metabolism of fatty acids through the glycolysis pathway, and Tohge et al.^88^ reported that this transferase played an important role in flavonoid biosynthesis. This study presented a dynamic picture of the maturation process of *C. oleifera* seeds on Hainan Island by using an exploratory multiomics dataset combined with WGCNA and correlation analysis. Some key candidate genes or proteins participating in flavonoid biosynthesis and fatty acid metabolism were screened out, and their temporal expression specificities were also revealed. Nevertheless, the specific conversion relationships and corresponding modulatory mechanisms of these abovementioned genes or proteins are worthy of further analysis and verification through genetic engineering techniques to lay a foundation for molecular breeding and cultivation of new varieties and to improve the quality of *C. oleifera* oil produced.

## Conclusions

In summary, the present study applied RNA-seq transcriptome analysis in conjunction with iTRAQ proteomics technology to probe the dynamic changes in reserve accumulation of *C. oleifera* seeds at different stages of maturity. In addition, we examined the potential regulatory mechanisms concerning the biosynthesis and metabolism of flavonoids and fatty acids. Many functional transcripts (16,530) and protein species (1228) were recognized to have a significantly changed pattern, among which 317 DAPs were covered by the transcriptomic results. The regulatory networks of important metabolites were discussed in combination with the expression profiles of structural genes (proteins) or TFs and the contents of corresponding compounds, revealing that the synthesis ability of flavonoids was attenuated during seed ripening, while that of fatty acids increased initially and then declined. We further identified two gene modules (indianred and tan2) and four protein modules (magenta, midnightblue, black, and yellow) related to flavonoid, oil, and fatty acid anabolism by using WGCNA. Notably, based on the known metabolic pathways and WGCNA combined with a correlation analysis, five coexpressed transcripts and proteins (CADs, COMT, FLS, and 4CL) were screened out, among which one member of interest (FLS) was selected for the bioinformatics assay. Finally, qRT-PCR validation indicated that our sequencing results were reliable. Consequently, the data provide a perspective for fully understanding the roles of genes and proteins that contribute to the oil quality of *C. oleifera* from Hainan Island. In addition, the screening of candidate genes or proteins that underwent remarkable variation in related pathways could serve as a foundation for marker-based breeding of other oil-pressing plants.

## Supplementary information

Supplementary Information

Table S1

Table S2

Table S3

Table S4

Table S5

Table S6

Table S7

Table S8

Table S9

Table S10

Table S11

Table S12

Table S13

Table S14

Table S15

Table S16

Table S17

Table S18

Figure S1

Figure S2

Figure S3

Figure S4

Figure S5

Figure S6

Figure S7

Figure S8

Figure S9

Figure S10

Figure S11

Figure S12

Figure S13

Figure S14

Figure S15

Figure S16

Figure S17

Figure S18

Figure S19

Figure S20

Figure S21

## Data Availability

The RNA-sequencing data were uploaded to the NCBI Sequence Read Archive (SRA, http://www.ncbi.nlm.nih.gov/sra) database, with the accession number PRJNA660557. The mass spectrometry proteomics data and spectra for modified peptides have been deposited to the ProteomeXchange Consortium (http://proteomecentral.proteomexchange.org) via the iProX partner repository with the dataset identifier PXD021909. The mass spectrometry proteomics data have also been deposited at iProX with the dataset identifier IPX0002528000.
