# Supplementary material for: Integrative iTRAQ-based proteomic and transcriptomic analysis reveals the accumulation patterns of key metabolites associated with oil quality during seed ripening of Camellia oleifera
Source: Hortic Res. 2021 Jul 1;8:157. doi: 10.1038/s41438-021-00591-2 (PMC8245520; doi:10.1038/s41438-021-00591-2)
Supplement: Supplementary file 1 — Supplementary Information [file 41438_2021_591_MOESM1_ESM.docx]

**Supplementary Information**

**Figures**

**Fig. S1 Total ionization chromatogram of fatty acid methyl ester standards.**

The numbers in this figure were as follows: 1, Methyl hexanoate (C6:0); 2, Methyl octanoate (C8:0); 3, Methyl decanoate (C10:0); 4, Methyl undecanoate (C11:0); 5, Methyl laurate (C12:0); 6, Methyl tridecanoate (C13:0); 7, Methyl myristate (C14:0); 8, Methyl myristoleate (C14:1n5); 9, Methyl pentadecanoate (C15:0); 10, Methyl pentadecenoate (C15:1n5); 11, Methyl palmitate (C16:0); 12, Methyl palmitoleate (C16:1n7); 13, Methyl heptadecanoater (C17:0); 14, Methyl heptadecenoate (C17:1n7); 15, Methyl stearate (C18:0); 16, Methyl elaidate (C18:1n9t); 17, Methyl oleate (C18:1n9c); 18, Methyl linolelaidate (C18:2n6t); 19, Methyl linoleate (C18:2n6c); 20, Methyl arachidate (C20:0); 21, Methyl γ-linolenate (C18:3n6); 22, Eicosenoic acid methyl ester (C20:1); 23, Methyl α-linolenate (C18:3n3); 24, Methyl heneicosanoate (C21:0); 25, Eicosadienoic acid methyl ester (C20:2); 26, Methyl behenate (C22:0); 27, cis-8,11,14-Eicosatrienoic acid methyl ester (C20:3n6); 28, Methyl erucate (C22:1n9); 29, cis-11,14,17-Eicosatrienoic acid methyl ester (C20:3n3); 30, Methyl arachidonate (C20:4n6); 31, Methyl tricosanoate (C23:0); 32, Docosadienoic acid methyl ester (C22:2n6); 33, Methyl tetracosanoate (C24:0); 34, Eicosapentaenoic acid methyl ester (C20:5n3); 35, Methyl tetracosenoate (C24:1n9); 36, Docosahexaenoic acid methyl ester (C22:6n3). Methyl butyrate (C4:0) was not detected.

**Fig. S2 Analysis of repeatability test of *C. oleifera* seed transcriptome.**

**Fig. S3 Overall functional annotation of assembled unigenes in *C*. *oleifera* seeds.**

Venn diagram showing the functional annotations of assembled unigenes coverage in the Nr, Nt, Swiss-Prot, Pfam, KO, KOG, and GO databases (**A**). Sequence homology characteristics of assembled unigenes against the NR database: the top eight species distribution (**B**). KOG function classification of assembled unigenes. Numbers and percentages of these unigenes in different clusters were summarized (**C**). Numbers and percentages of these unigenes in different clusters were summarized, and the abbreviations were as follows: A, RNA processing and modification; B, Chromatin structure and dynamics; C, Energy production and conversion; D, Cell cycle control, cell division, chromosome partitioning; E, Amino acid transport and metabolism; F, Nucleotide transport and metabolism; G, Carbohydrate transport and metabolism; H, Coenzyme transport and metabolism; I, Lipid transport and metabolism; J, Translation, ribosomal structure and biogenesis; K, Transcription; L, Replication, recombination and repair; M, Cell wall/membrane/envelope biogenesis; N, Cell motility; O, Posttranslational modification, protein turnover, chaperones; P, Inorganic ion transport and metabolism; Q, Carbohydrate transport and metabolism; R, General function prediction only; S, Function unknown; T, Signal transduction mechanisms; U, Intracellular trafficking, secretion, and vesicular transport; V, Defense mechanisms; W, Extracellular structures; Y, Nuclear structure; Z, Cytoskeleton.

**Fig. S4 GO classification of the assembled unigenes in *C. oleifera* seeds.**

**Fig. S5 KEGG pathway analysis of the assembled unigenes in *C. oleifera* seeds.**

**Fig. S6 GO enrichment analysis of the identified DEGs in developing *C*. *oleifera* seeds.**

The three comparison groups included S2 vs. S1 (**A**), S3 vs. S1 (**B**) and S4 vs. S1 (**C**).

**Fig. S7 Top KEGG pathway enrichment analysis of the identified DEGs in developing *C*. *oleifera* seeds.**

The rich factor is the percentage of genes out of the total number of detected genes in the KEGG pathway. The bubble size represents the number of unigenes detected in KEGG pathway, and the color of bubble represents *q*-value, and the *q*-value is equal to the corrected *p*-value in the enrichment analysis. The three comparison groups included S2 vs. S1 (**A**), S3 vs. S1 (**B**) and S4 vs. S1 (**C**).

**Fig. S8** **Heat map and eigengene expression profile for the WGCNA modules in developing *C*. *oleifera* seeds.**

The heatmap shows gene expression level in all samples of ‘indianred’ (**A**) and ‘tan2’ (**B**) modules. Red denotes over-expression, green under-expression and bar plot represents eigengene of samples.

**Fig. S9 Functional annotation of the identified DEGs related to flavonoid and oil anabolism in developing *C. oleifera* seeds.**

GO enrichment analysis of these DEGs (**A**). KEGG pathway enrichment categories of theses DEGs (**B**). Protein-protein interactions of these DEGs (**C**).

**Fig. S10 The SDS-PAGE and overall functional annotation of expressed proteins in *C*. *oleifera* seeds.**

SDS-PAGE gel map of extracted proteins with three biological repeats (**A**). Sequence homology characteristics of expressed proteins against the NR database: the top eight species distribution (**B**). KOG function classification of expressed proteins. Numbers and percentages of the proteins in different clusters were presented (**C**). Numbers and percentages of the proteins in different clusters were presented, and the abbreviations were as follows: A, RNA processing and modification; B, Chromatin structure and dynamics; C, Energy production and conversion; D, Cell cycle control, cell division, chromosome partitioning; E, Amino acid transport and metabolism; F, Nucleotide transport and metabolism; G, Carbohydrate transport and metabolism; H, Coenzyme transport and metabolism; I, Lipid transport and metabolism; J, Translation, ribosomal structure and biogenesis; K, Transcription; L, Replication, recombination and repair; M, Cell wall/membrane/envelope biogenesis; N, Cell motility; O, Posttranslational modification, protein turnover, chaperones; P, Inorganic ion transport and metabolism; Q, Carbohydrate transport and metabolism; R, General function prediction only; S, Function unknown; T, Signal transduction mechanisms; U, Intracellular trafficking, secretion, and vesicular transport; V, Defense mechanisms; Y, Nuclear structure; Z, Cytoskeleton.

**Fig. S11 GO classification of the recognized proteins and DAPs in developing *C*. *oleifera* seeds.**

Numbers represent the proportion of proteins annotated to the GO terms. Blue bars represent the all proteins, and red bars represent the differentially expressed ones. The three comparison groups included S2 vs. S1 (**A**), S3 vs. S1 (**B**) and S4 vs. S1 (**C**).

**Fig. S12 KEGG pathway analysis of the recognized proteins and DAPs in developing *C*. *oleifera* seeds.**

The KEGG metabolic pathway names are presented on the y-axis. Numbers represent the proportion of proteins annotated to the pathway. Grey bars represent the all proteins, and yellow bars represent the differentially expressed ones. The three comparison groups included S2 vs. S1 (**A**), S3 vs. S1 (**B**) and S4 vs. S1 (**C**).

**Fig. S13 Top KEGG pathway enrichment analysis of the recognized DAPs in developing *C*. *oleifera* seeds.**

The rich factor is the percentage of proteins out of the total number of detected proteins in the KEGG pathway. The bubble size represents the number of proteins detected in KEGG pathway, and the color of bubble represents *p-value*. The three comparison groups included S2 vs. S1 (**A**), S3 vs. S1 (**B**) and S4 vs. S1 (**C**).

**Fig. S14** **Heat map and eigengene expression profile for the WGCNA modules in developing *C*. *oleifera* seeds.**

The heatmap shows protein expression level in all samples of ‘magenta’ (**A**), ‘midnightblue’ (**B**), ‘black’ (**C**), and ‘yellow’ (**D**) modules. Red denotes over-expression, green under-expression and bar plot represents eigengene of samples.

**Fig. S15 Functional annotation of the identified DAPs related to flavonoid and oil anabolism in developing *C. oleifera* seeds.**

GO enrichment analysis of these DAPs (**A**). KEGG pathway enrichment categories of theses DAPs (**B**). Protein-protein interactions of these DAPs (**C**).

**Fig. S16 Venn diagram of the co-expressive DEGs and DAPs abundance changes of S2 vs. S1 (A), S3 vs. S1 (B) and S4 vs. S1 (C) groups.**

Venn diagram reveals the cognate DEGs and DAPs that were consistently up-regulated (**D**) and down-regulated (**E**) in three comparative analyses.

**Fig. S17** **Correlation between mRNA and protein abundance changes in developing *C*. *oleifera* seeds.**

Volcano plots for mRNAs and proteins in three pairwise comparisons of S2 vs. S1 (a), S3 vs. S1 (b) and S4 vs. S1 (c). The green and red points represent the unigenes and proteins with the same or opposite expression trends, respectively (A). Distribution of Spearman’s correlation coefﬁcients of mRNAs and proteins in three comparative groups of S2 vs. S1 (a), S2 vs. S1 (b) and S3 vs. S1 (c). Blue, positive correlations (B).

**Fig. S18 Hierarchical clustering analysis of the identified DEGs in four growth periods of seeds.**

Red color indicates high expression of gene or protein, and blue color indicates low expression of gene or protein. The three comparison groups included S2 vs. S1 (**A**), S3 vs. S1 (**B**) and S4 vs. S1 (**C**).

**Fig. S19 Double Pie charts for GO functional classification of the co-expressive DEGs and DAPs of S2 vs. S1 (A), S3 vs. S1 (B) and S4 vs. S1 (C) groups.**

The proportion of each category which include biological process, molecular function and cellular component displayed is based on in percentage. The inner ring is the distribution of differential mRNA, and the outer ring is the distribution of differential proteins.

**Fig. S20 Motif analysis of FLS proteins from different plants.**

The sequence alignment of SRG1 and FLS proteins. *Camellia sinensis* (ARM53419.1), *Camellia fraterna* (AUM57439.1), *Cyclamen purpurascens* (BBA27023.1), *Rhododendron simsii* (KAF7114282.1), *Camellia nitidissima* (ADZ28516.1), *Nyssa sinensis* (KAA8546373.1), Vitis vinifera (VvFLS, BAE75810.1) (**A**). Motif sequences of these FLS proteins (**B**).

**Fig. S21 The relative sequence features and phylogenetic analysis of the FLS protein.**

Multiple sequence alignment of the FLS proteins was performed using DNAMAN (**A**). Phylogenetic analysis of the FLS proteins in diferent species (**B**).

**Tables**

Table S1 Linear regression data of fatty acid methyl ester standards.

Table S2 Summary of the sequencing data from different *C. oleifera* seeds.

Table S3 Functional annotation of the assembled unigenes in different databases.

Table S4 The annotations and classifications by three independent RNA-Seq transcriptome analyses of assembled unigenes.

Table S5 The information of the identified DEGs in developing *C*. *oleifera* seeds.

Table S6 GO terms and KEGG enrichment pathways of the identified DEGs including TFs in developing *C*. *oleifera* seeds.

Table S7 Protein-protein interactions of the identified DEGs in developing *C*. *oleifera* seeds.

Table S8 The WGCNA information of the identified genes in developing *C*. *oleifera* seeds.

Table S9 The correlation information of the identified DEGs related to flavonoid and oil anabolism in developing *C*. *oleifera* seeds.

Table S10 The identification information and functional annotation of the recognized proteins in developing *C*. *oleifera* seeds.

Table S11 The information of the recognized DAPs in developing *C*. *oleifera* seeds.

Table S12 GO terms and KEGG enrichment pathways of the expressed proteins and DAPs including TFs in developing *C*. *oleifera* seeds.

Table S13 Protein-protein interactions of the recognized DAPs in developing *C*. *oleifera* seeds.

Table S14 The WGCNA information of the recognized proteins in developing *C*. *oleifera* seeds.

Table S15 The correlation information of the recognized DAPs related to flavonoid and oil anabolism in developing *C*. *oleifera* seeds.

Table S16 The information of the correlated DEGs and DAPs in developing *C*. *oleifera* seeds.

Table S17 GO terms and KEGG enrichment pathways of the correlated DEGs and DAPs in developing *C*. *oleifera* seeds.

Table S18 Relative expression information of genes encoding target proteins in *C. oleifera* seeds by qRT-PCR assay.
