# Supplementary figures and images for: Integrative iTRAQ-based proteomic and transcriptomic analysis reveals the accumulation patterns of key metabolites associated with oil quality during seed ripening of Camellia oleifera
Source: Hortic Res. 2021 Jul 1;8:157. doi: 10.1038/s41438-021-00591-2 (PMC8245520; doi:10.1038/s41438-021-00591-2)

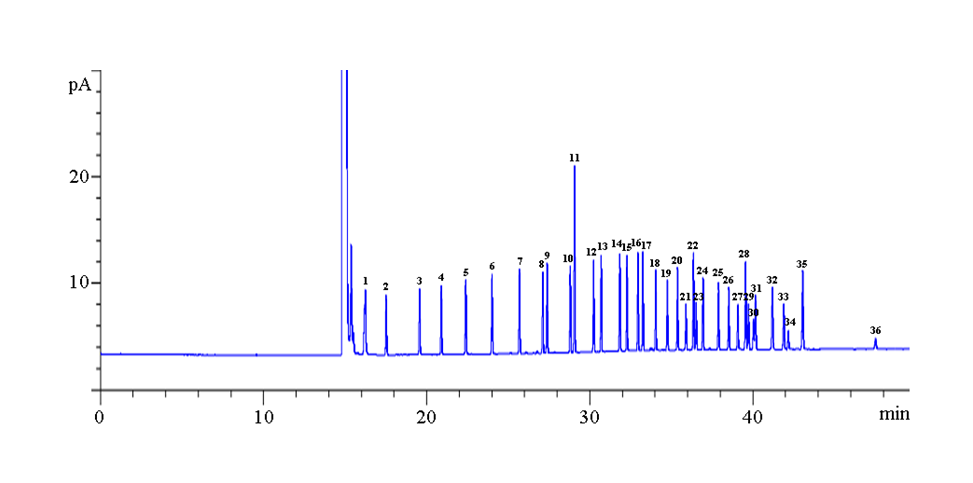

Supplement: Supplementary file 20 — Figure S1 [file 41438_2021_591_MOESM20_ESM.tif]

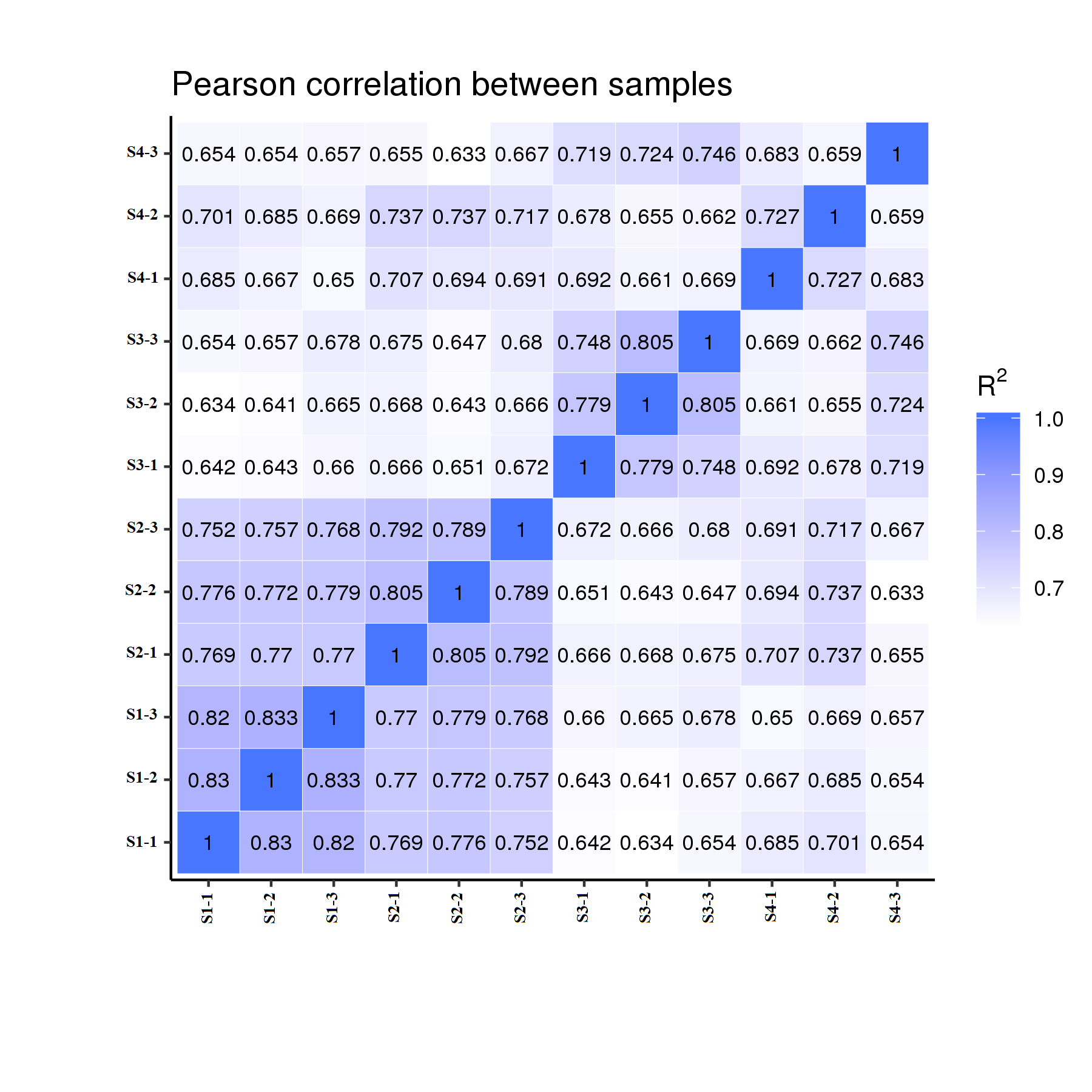

Supplement: Supplementary file 21 — Figure S2 [file 41438_2021_591_MOESM21_ESM.tif]

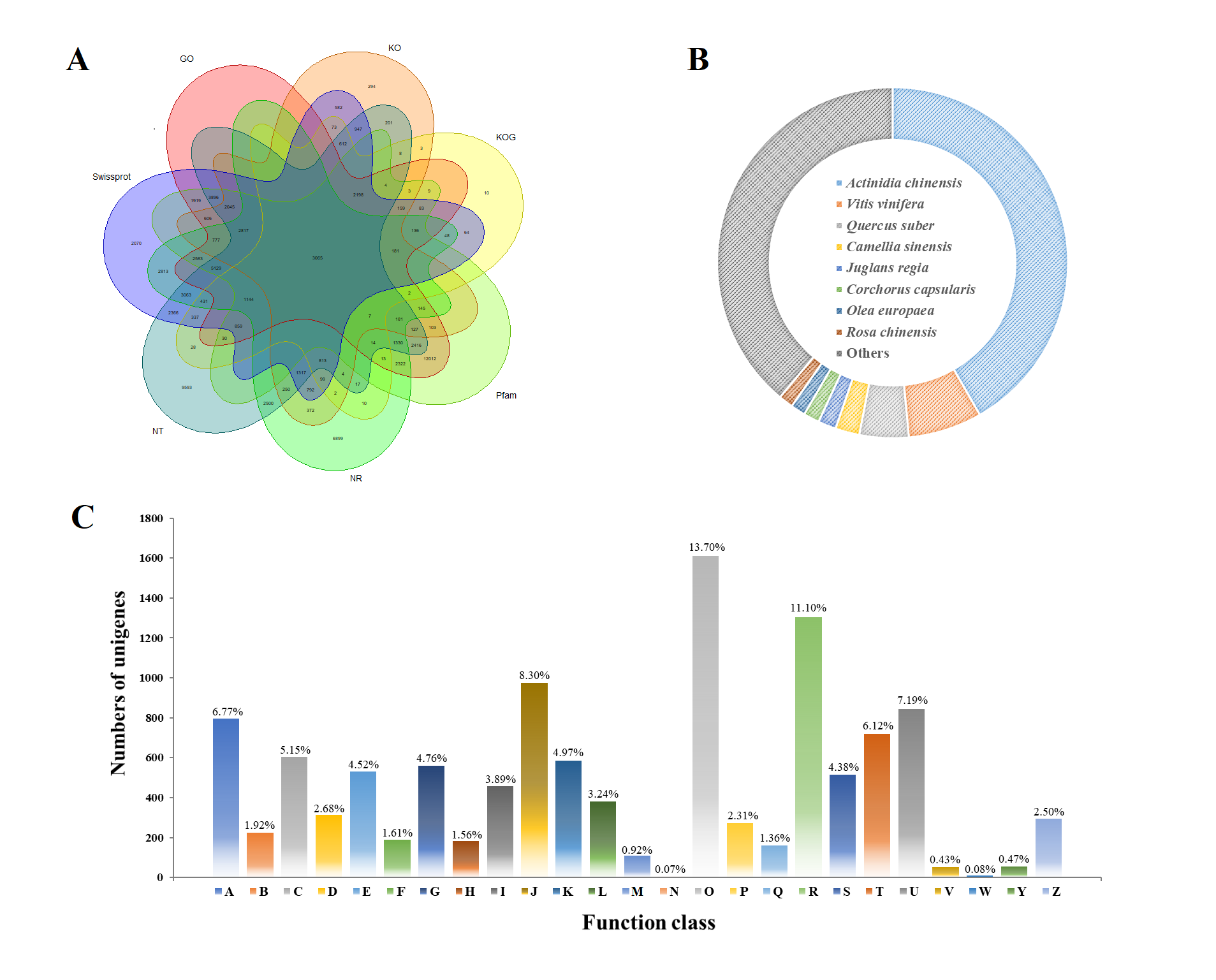

Supplement: Supplementary file 22 — Figure S3 [file 41438_2021_591_MOESM22_ESM.tif]

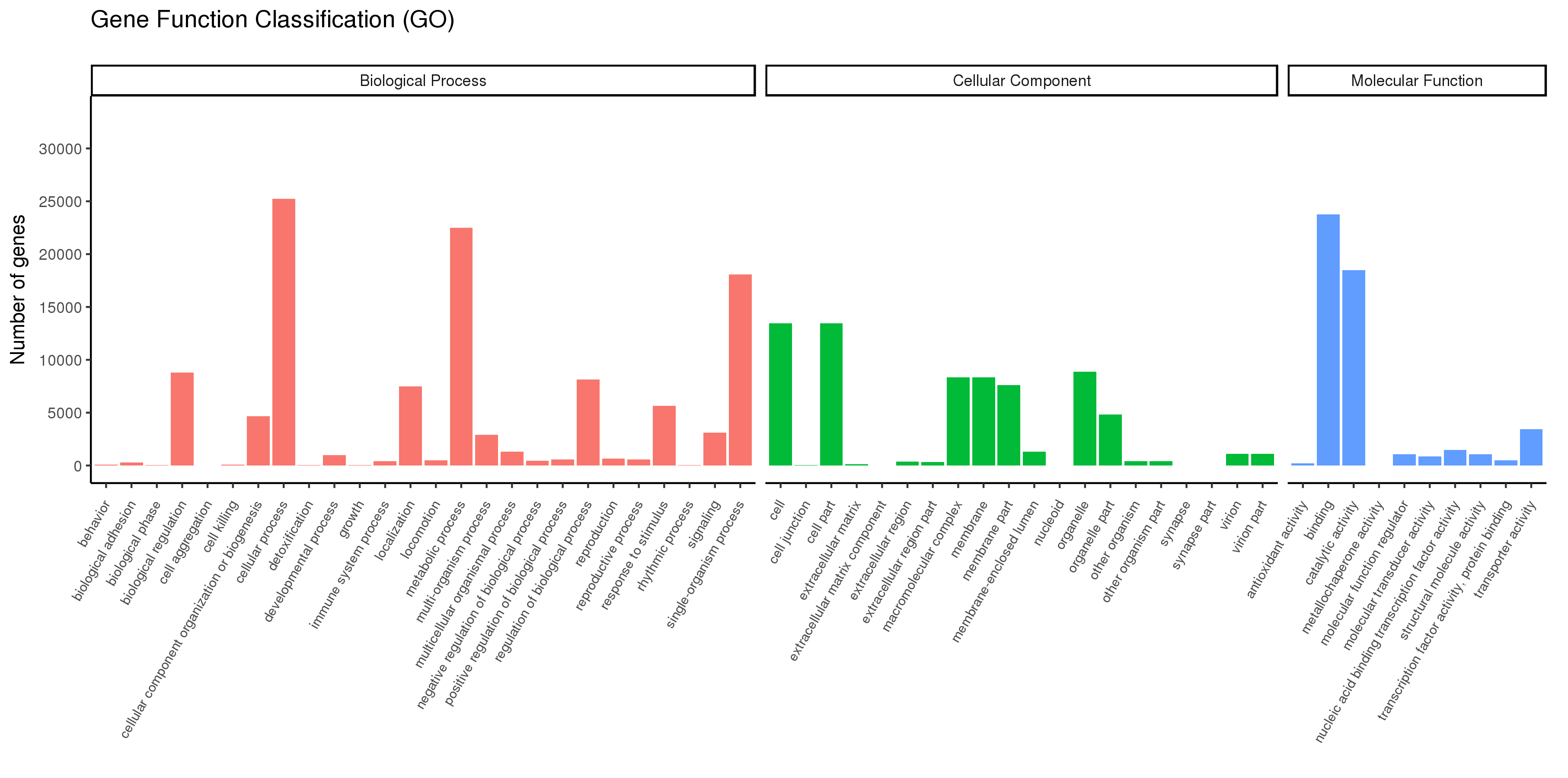

Supplement: Supplementary file 23 — Figure S4 [file 41438_2021_591_MOESM23_ESM.tif]

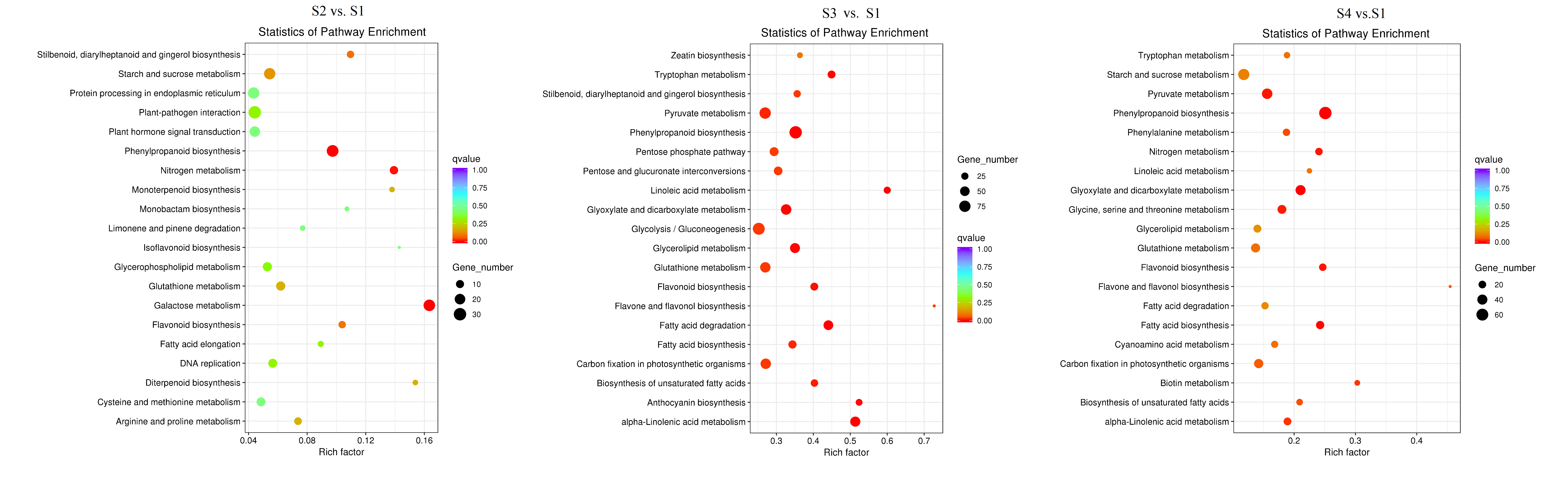

Supplement: Supplementary file 24 — Figure S5 [file 41438_2021_591_MOESM24_ESM.tif]

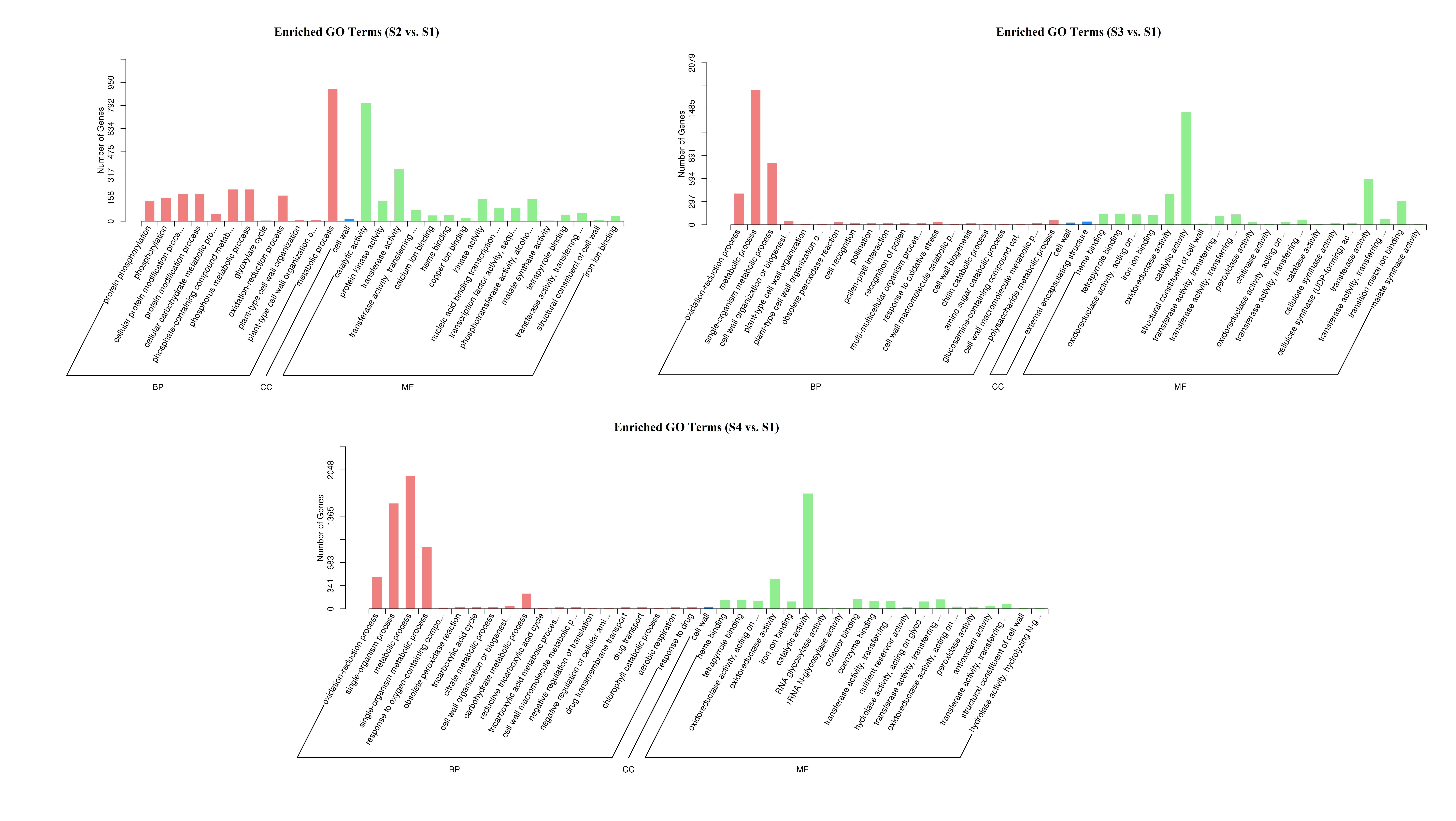

Supplement: Supplementary file 25 — Figure S6 [file 41438_2021_591_MOESM25_ESM.tif]

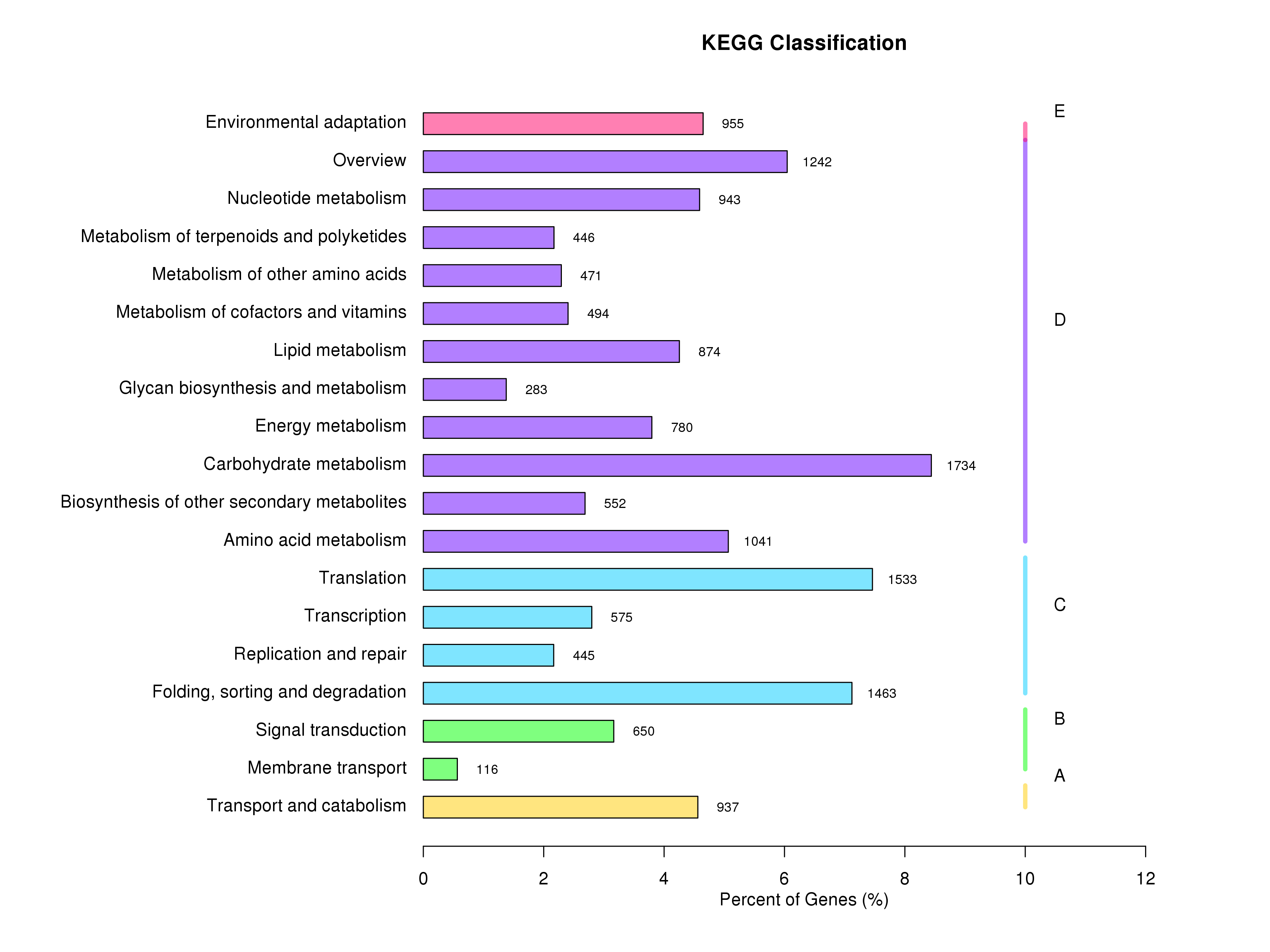

Supplement: Supplementary file 26 — Figure S7 [file 41438_2021_591_MOESM26_ESM.tif]

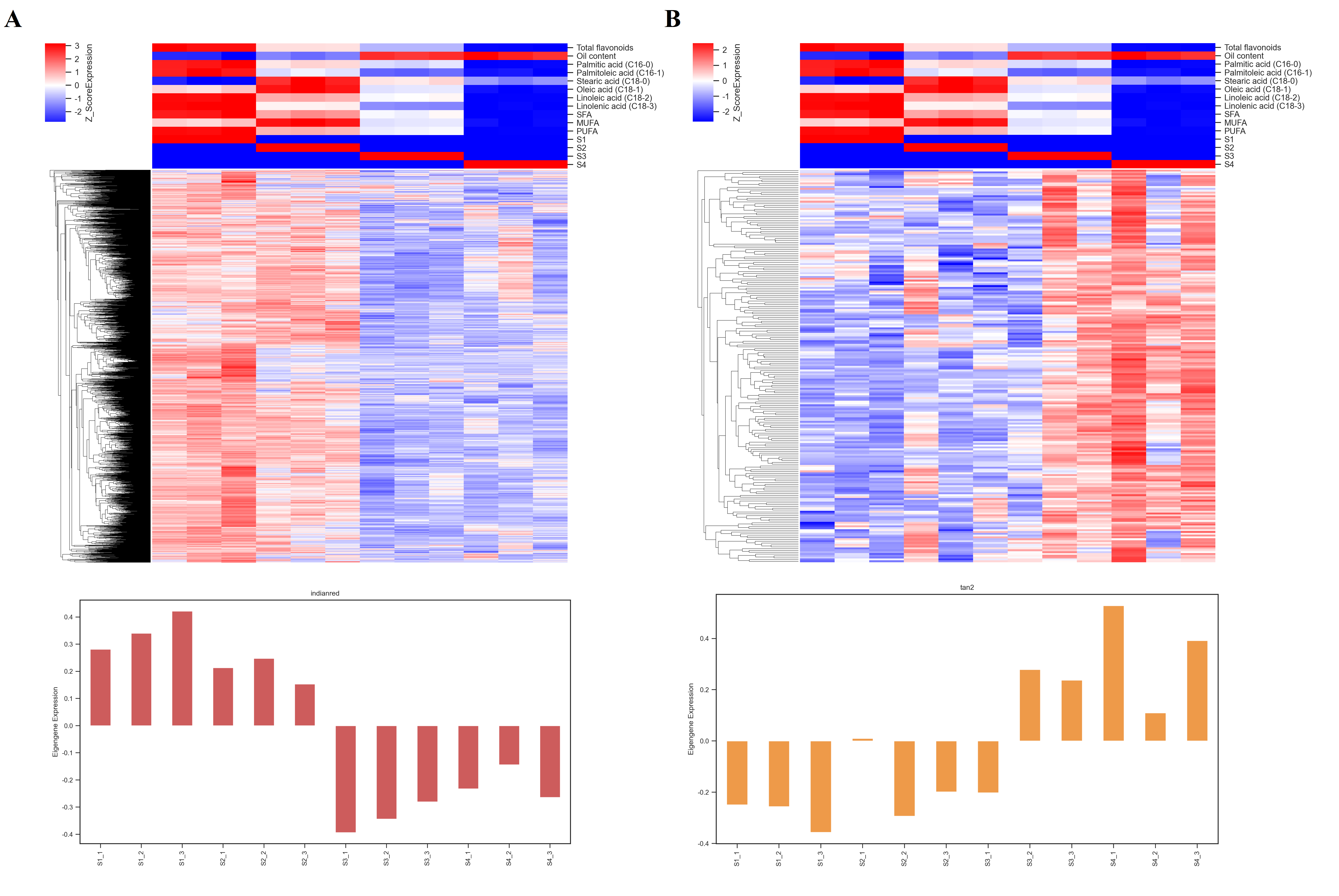

Supplement: Supplementary file 27 — Figure S8 [file 41438_2021_591_MOESM27_ESM.tif]

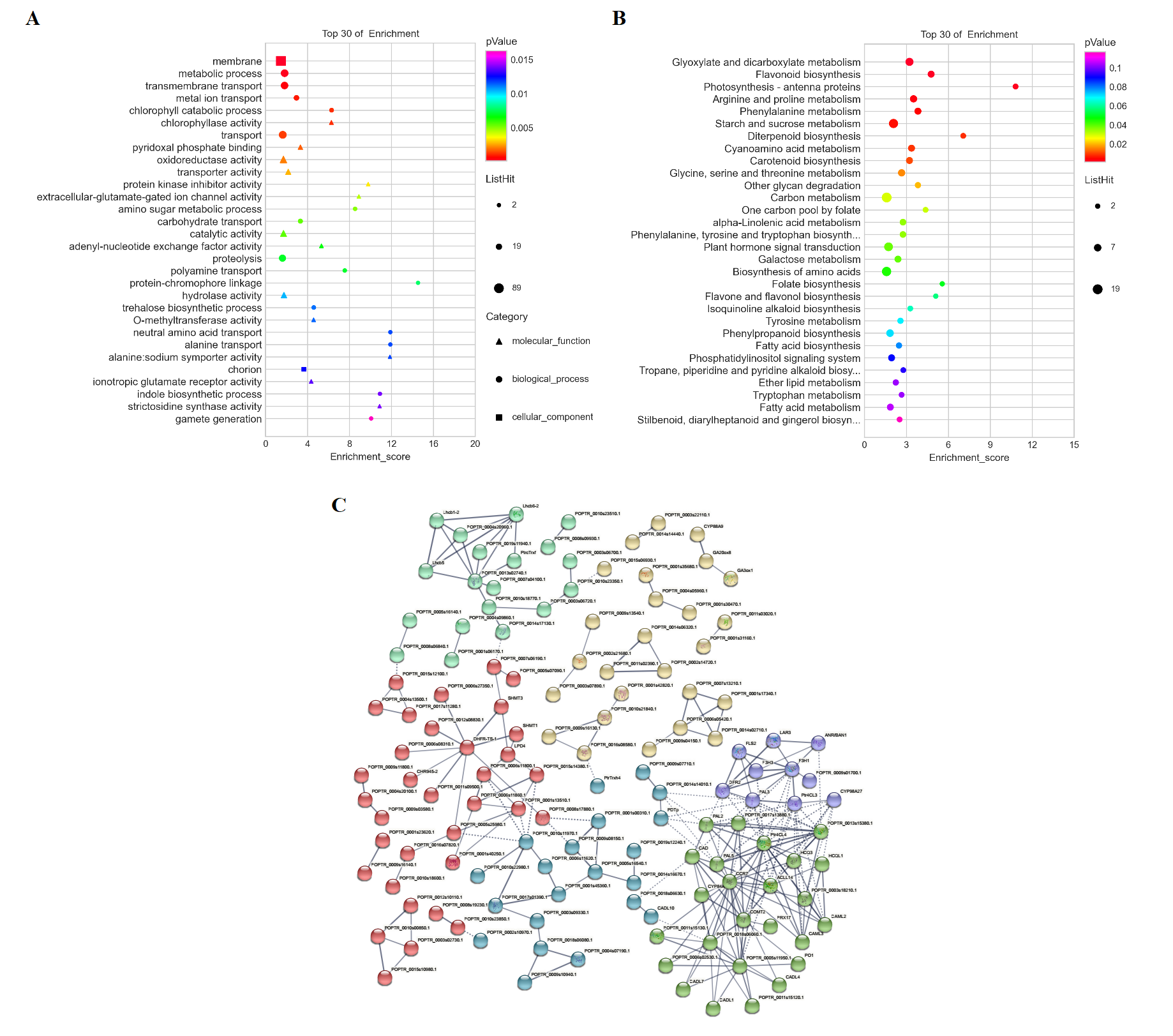

Supplement: Supplementary file 28 — Figure S9 [file 41438_2021_591_MOESM28_ESM.tif]

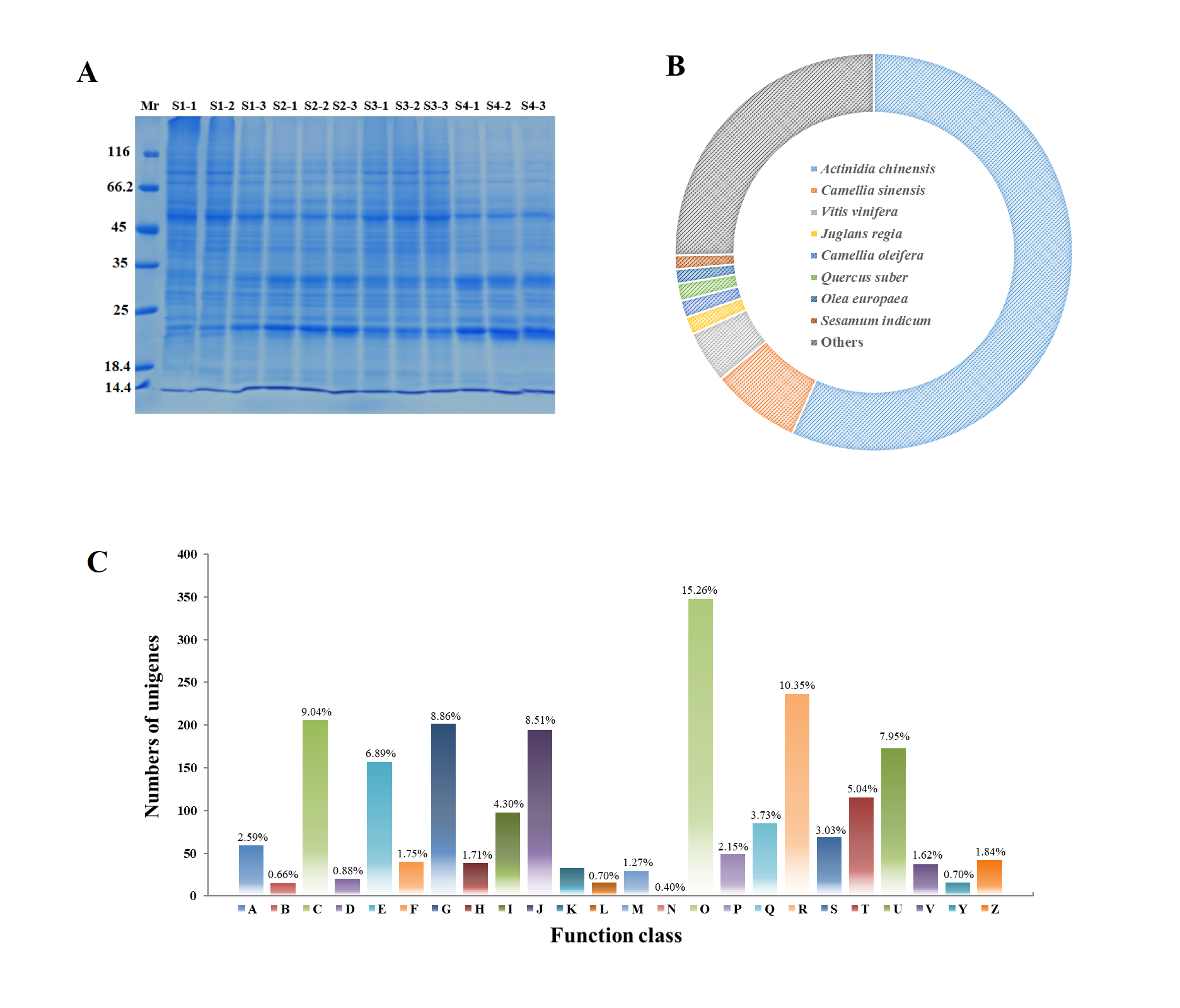

Supplement: Supplementary file 29 — Figure S10 [file 41438_2021_591_MOESM29_ESM.tif]

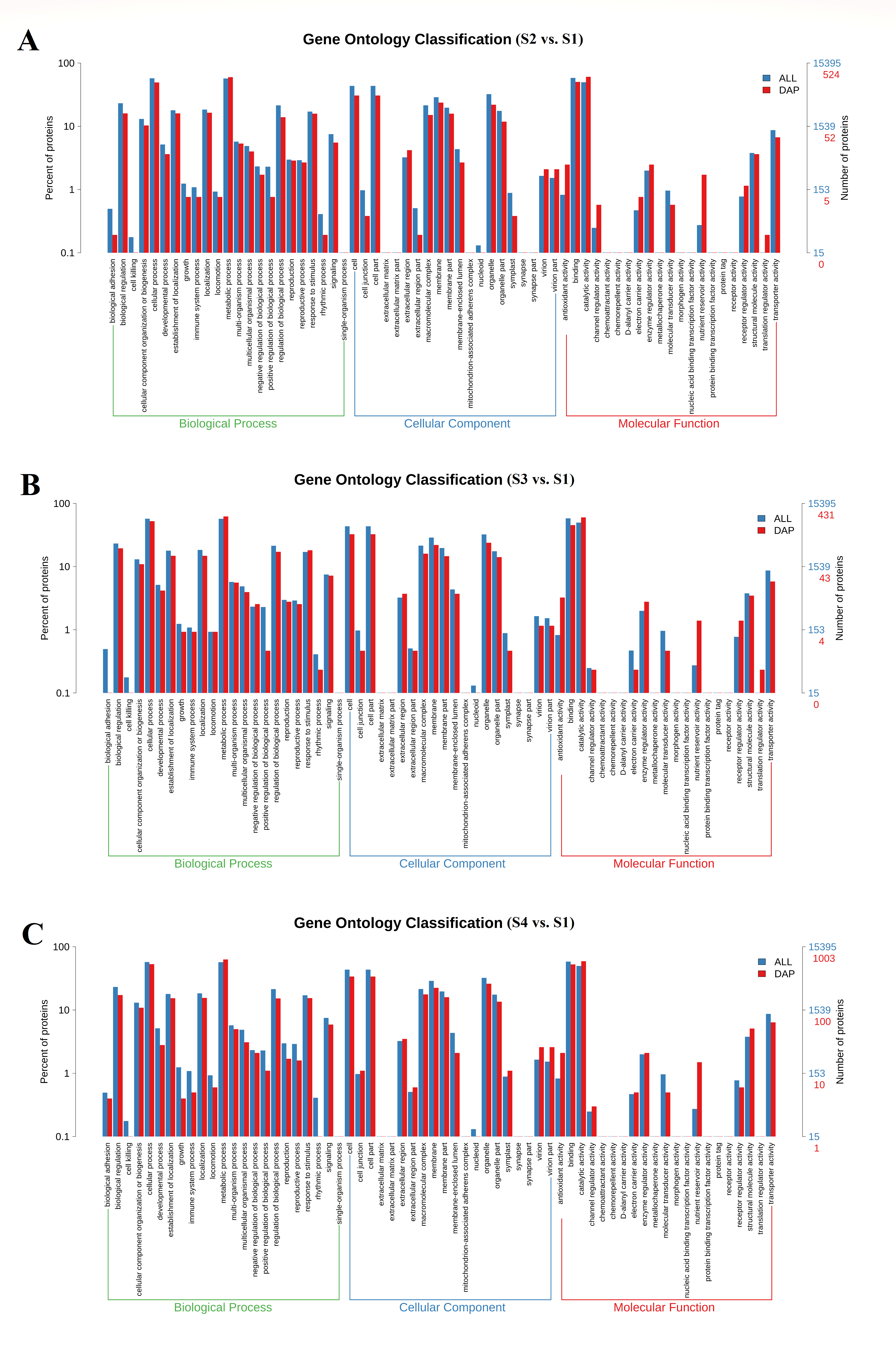

Supplement: Supplementary file 30 — Figure S11 [file 41438_2021_591_MOESM30_ESM.tif]

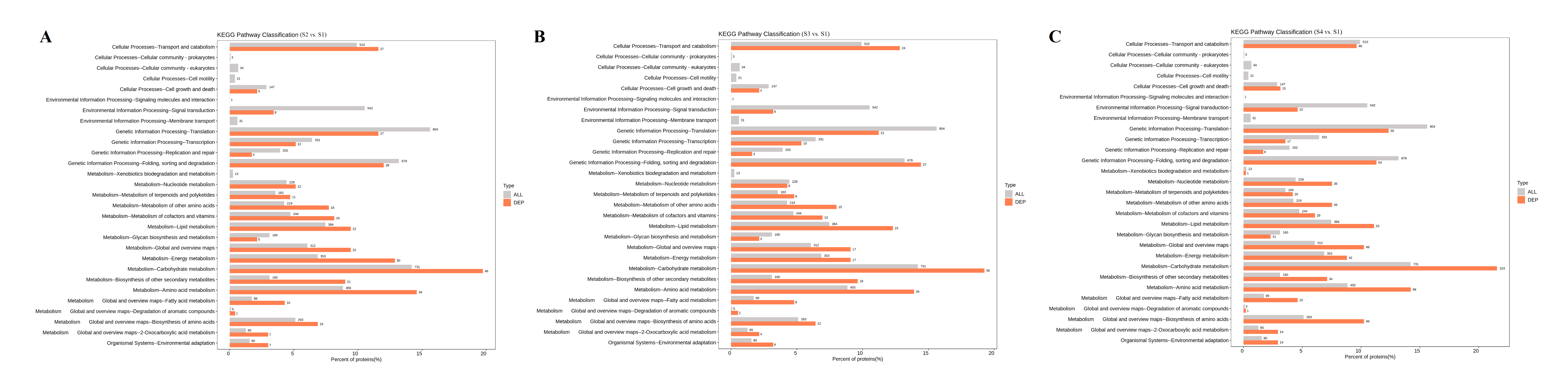

Supplement: Supplementary file 31 — Figure S12 [file 41438_2021_591_MOESM31_ESM.tif]

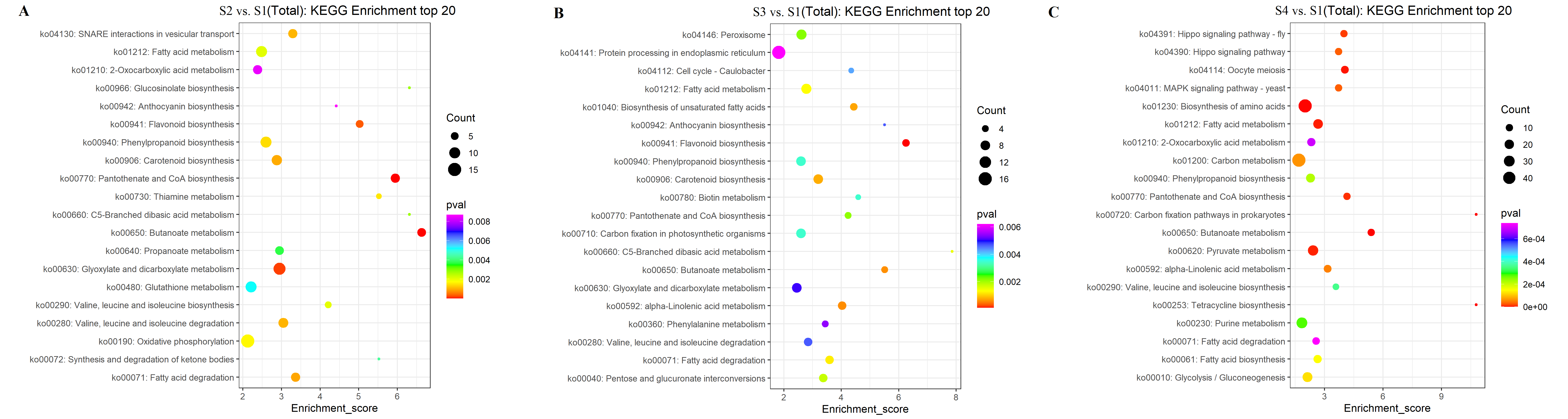

Supplement: Supplementary file 32 — Figure S13 [file 41438_2021_591_MOESM32_ESM.tif]

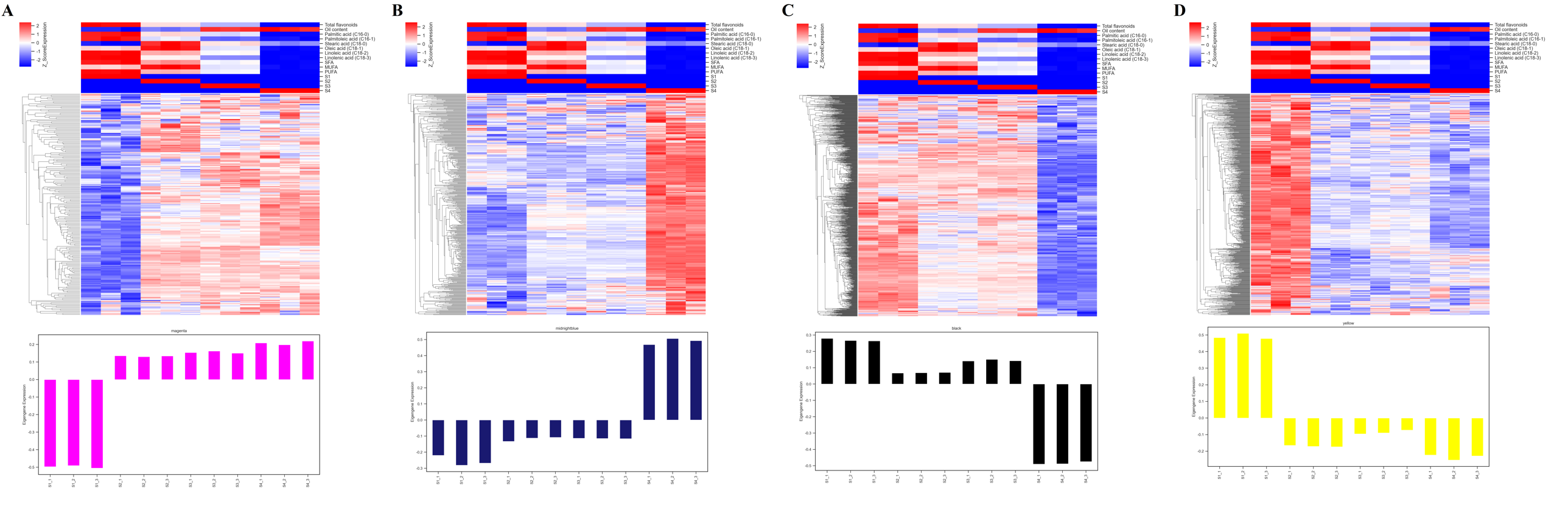

Supplement: Supplementary file 33 — Figure S14 [file 41438_2021_591_MOESM33_ESM.tif]

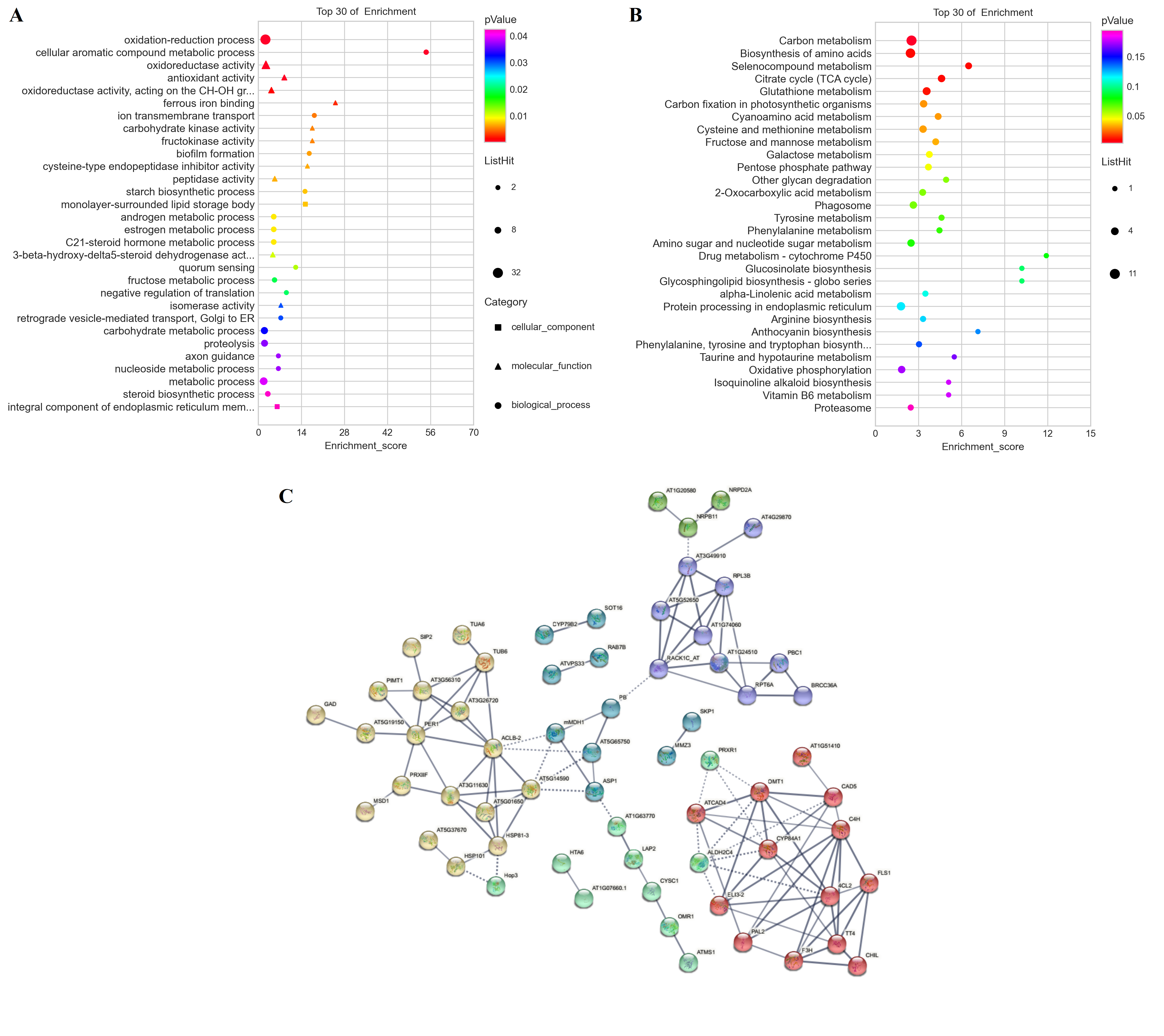

Supplement: Supplementary file 34 — Figure S15 [file 41438_2021_591_MOESM34_ESM.tif]

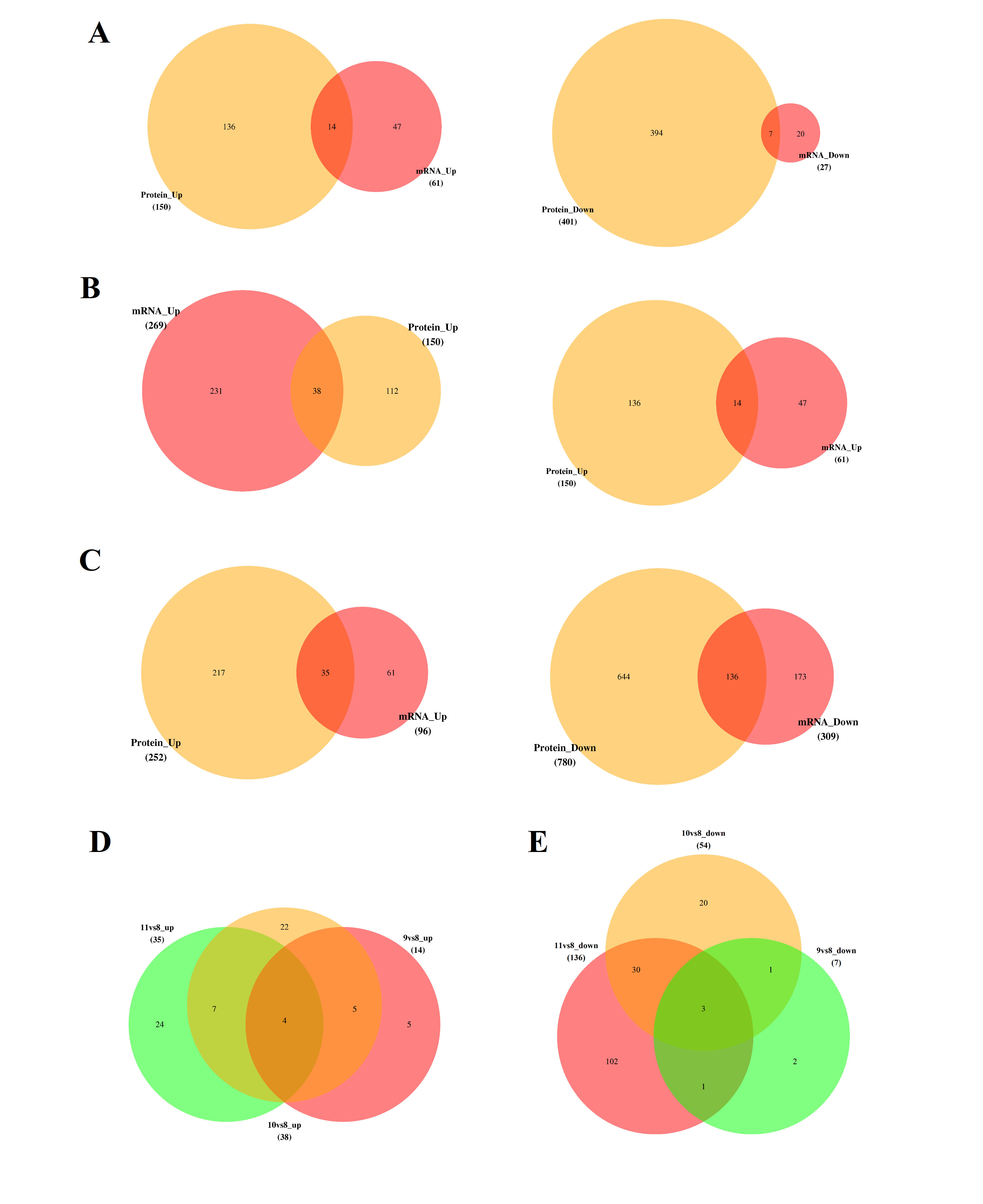

Supplement: Supplementary file 35 — Figure S16 [file 41438_2021_591_MOESM35_ESM.tif]

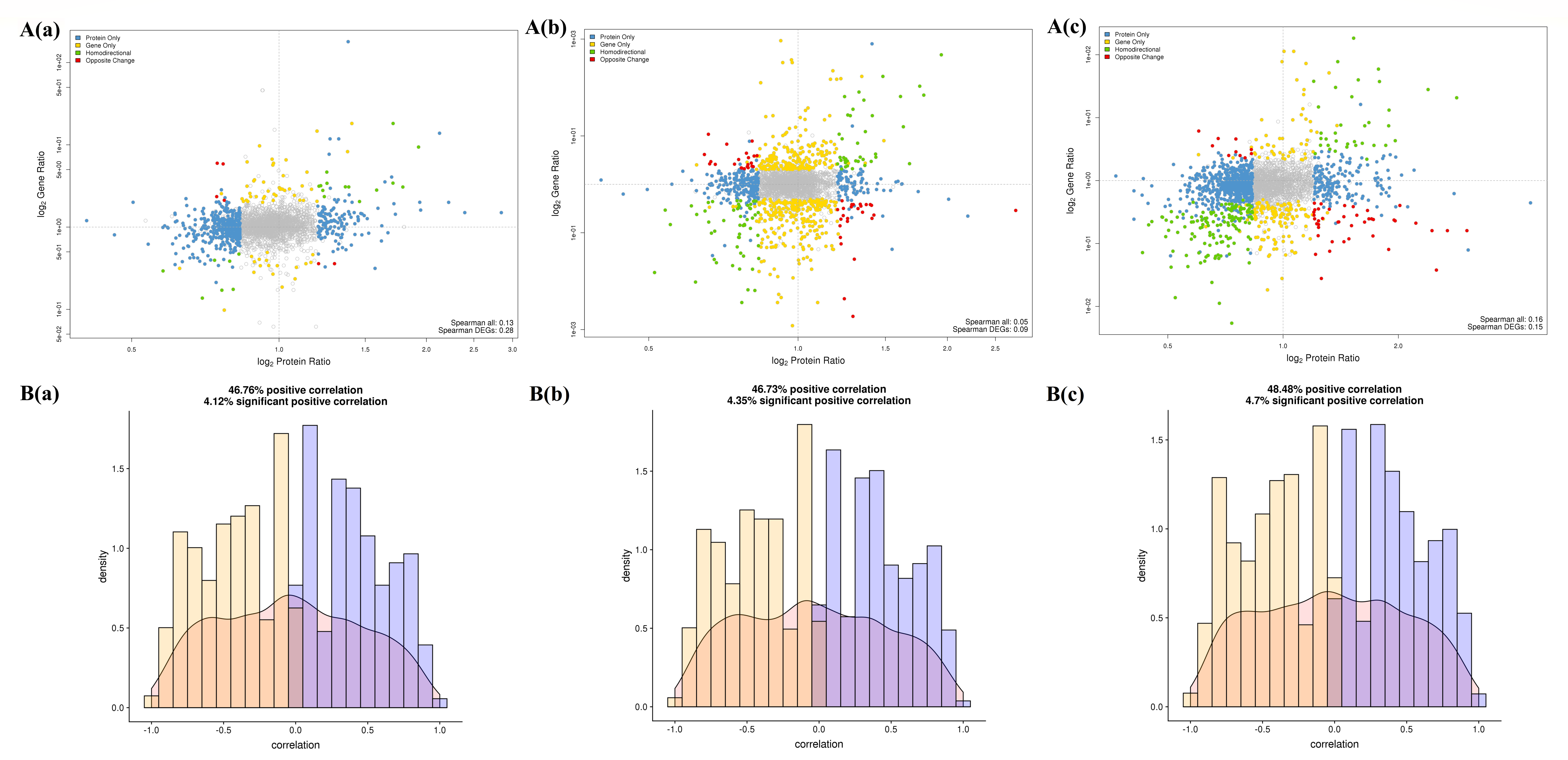

Supplement: Supplementary file 36 — Figure S17 [file 41438_2021_591_MOESM36_ESM.tif]

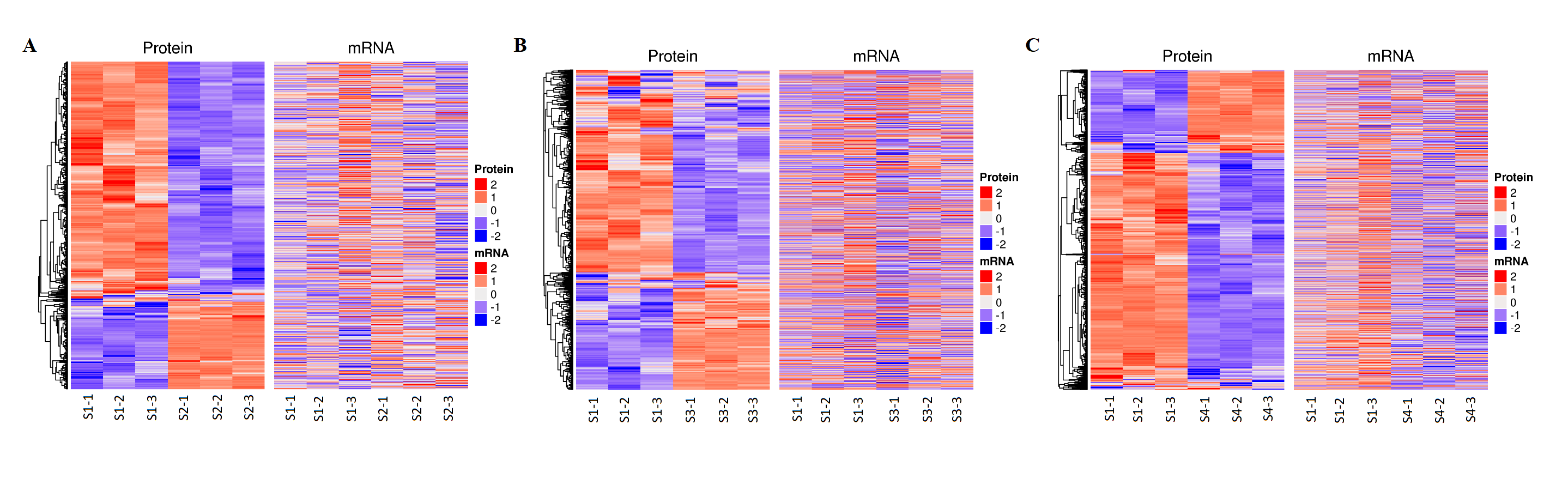

Supplement: Supplementary file 37 — Figure S18 [file 41438_2021_591_MOESM37_ESM.tif]

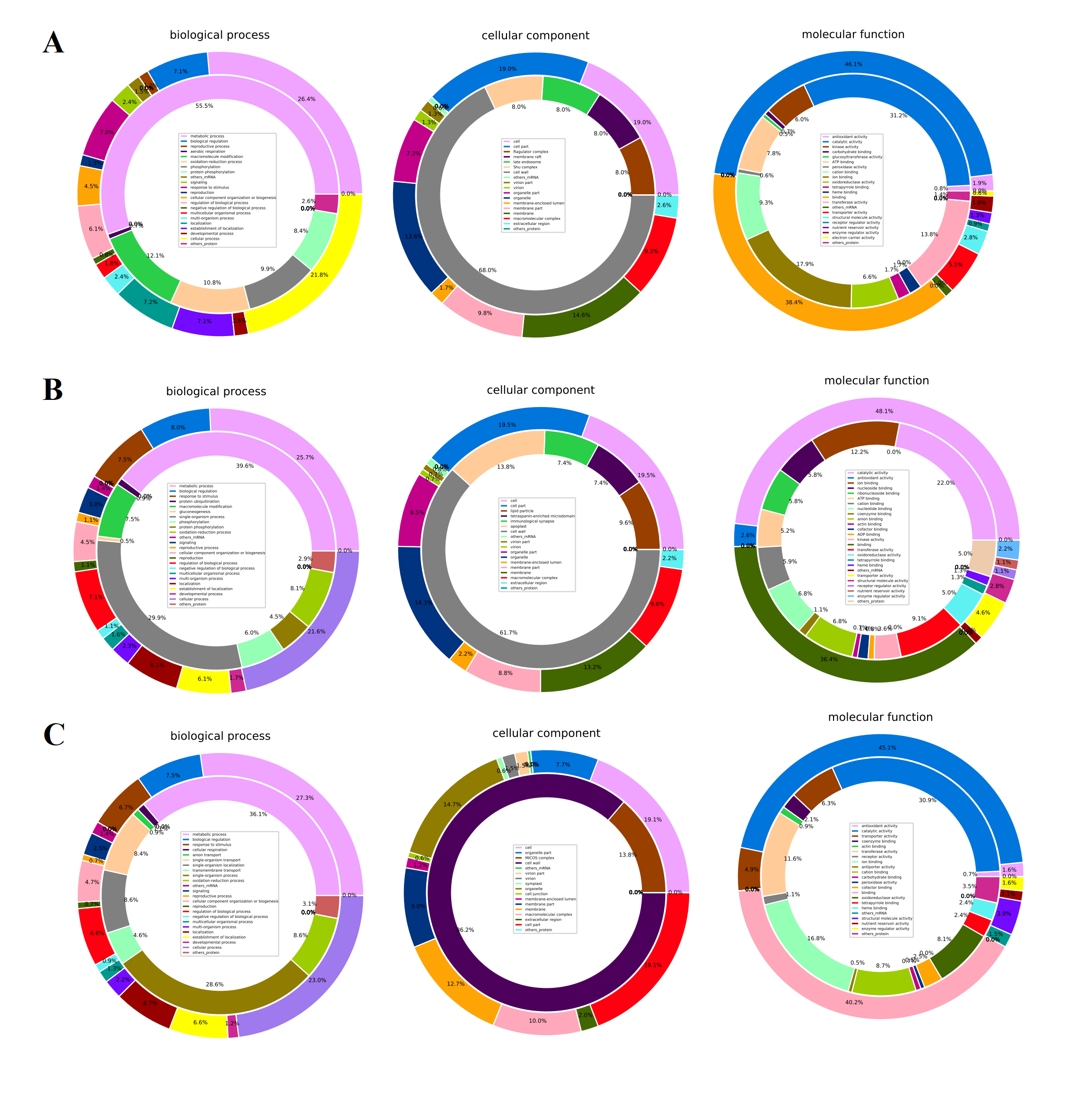

Supplement: Supplementary file 38 — Figure S19 [file 41438_2021_591_MOESM38_ESM.tif]

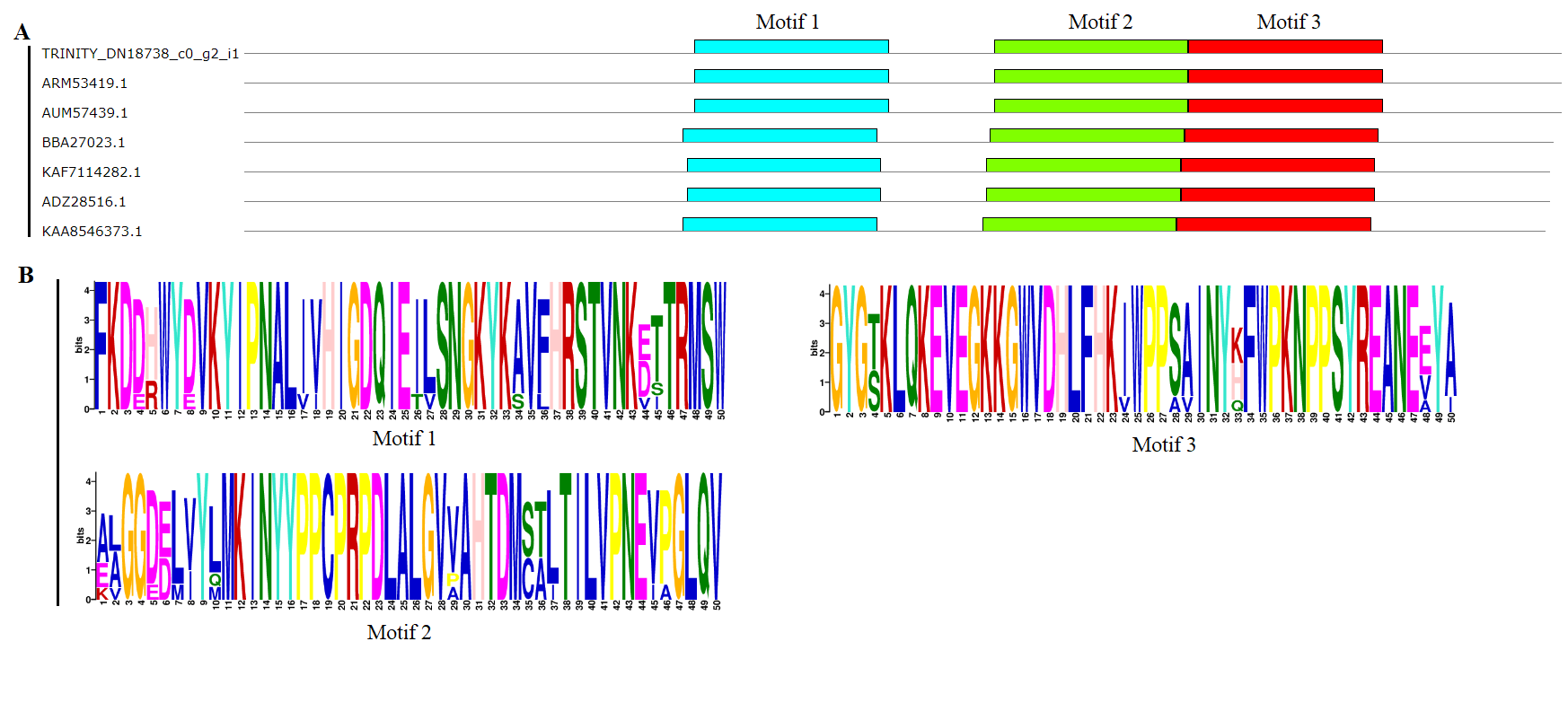

Supplement: Supplementary file 39 — Figure S20 [file 41438_2021_591_MOESM39_ESM.tif]

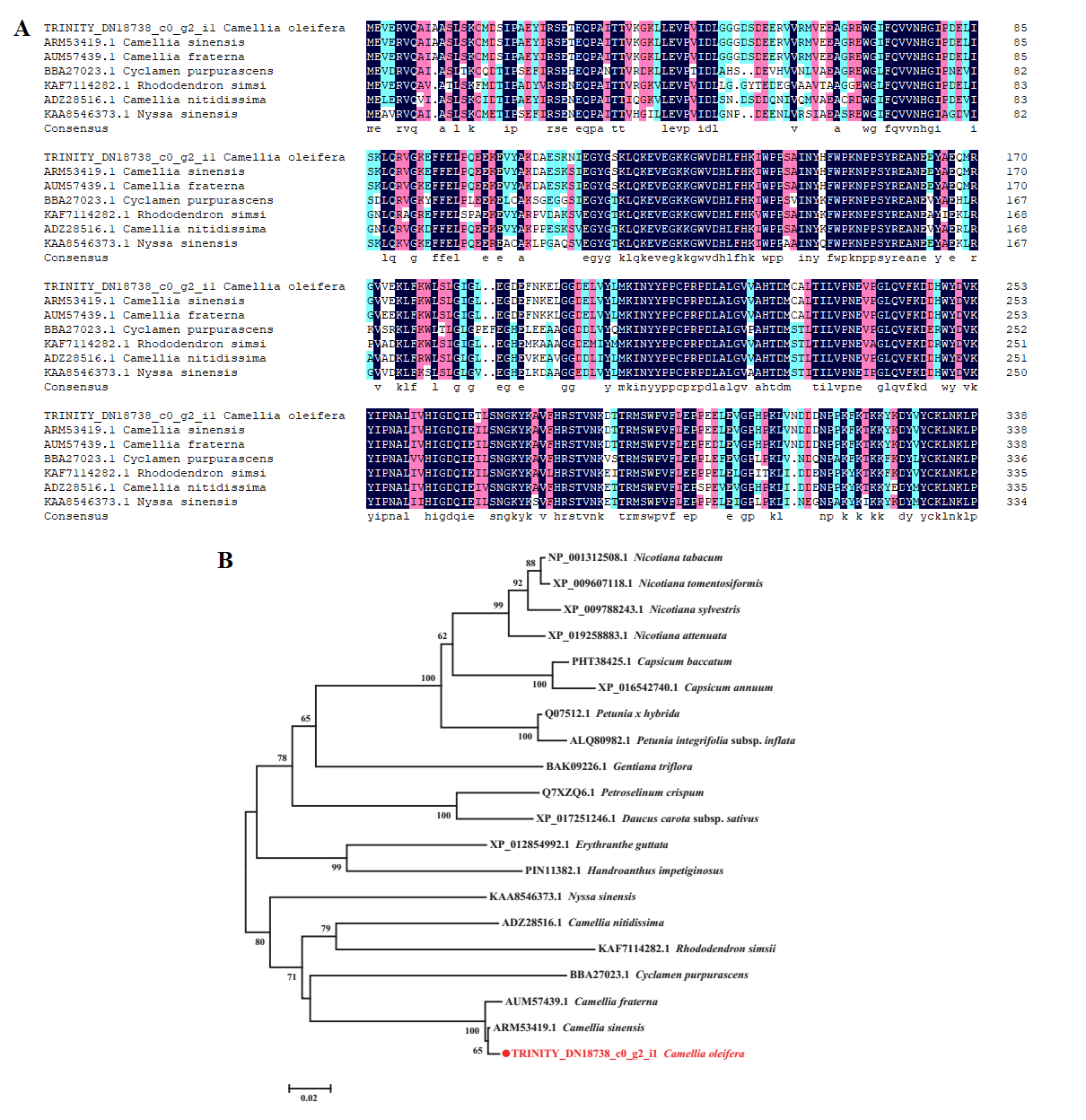

Supplement: Supplementary file 40 — Figure S21 [file 41438_2021_591_MOESM40_ESM.tif]
